# SHER: A Colored Petri Net Based Random Mobility Model for Wireless Communications

**DOI:** 10.1371/journal.pone.0133634

**Published:** 2015-08-12

**Authors:** Naeem Akhtar Khan, Farooq Ahmad, Sher Afzal Khan

**Affiliations:** 1 Faculty of Information Technology, University of Central Punjab, Lahore, Pakistan; 2 Department of Computer Science, COMSATS Institute of information Technology, Lahore, Pakistan; 3 Department of Computer Sciences, Abdul Wali Khan University, Mardan, Pakistan; 4 Faculty of Computing and Information Technology in Rabigh, King Abdul Aziz University, Jeddah, KSA; Nankai University, CHINA

## Abstract

In wireless network research, simulation is the most imperative technique to investigate the network’s behavior and validation. Wireless networks typically consist of mobile hosts; therefore, the degree of validation is influenced by the underlying mobility model, and synthetic models are implemented in simulators because real life traces are not widely available. In wireless communications, mobility is an integral part while the key role of a mobility model is to mimic the real life traveling patterns to study. The performance of routing protocols and mobility management strategies e.g. paging, registration and handoff is highly dependent to the selected mobility model. In this paper, we devise and evaluate the Show Home and Exclusive Regions (SHER), a novel two-dimensional (2-D) Colored Petri net (CPN) based formal random mobility model, which exhibits sociological behavior of a user. The model captures hotspots where a user frequently visits and spends time. Our solution eliminates six key issues of the random mobility models, i.e., *sudden stops*, *memoryless movements*, *border effect*, *temporal dependency of velocity*, *pause time dependency*, *and speed decay* in a single model. The proposed model is able to predict the future location of a mobile user and ultimately improves the performance of wireless communication networks. The model follows a uniform nodal distribution and is a mini simulator, which exhibits interesting mobility patterns. The model is also helpful to those who are not familiar with the formal modeling, and users can extract meaningful information with a single mouse-click. It is noteworthy that capturing dynamic mobility patterns through CPN is the most challenging and virulent activity of the presented research. Statistical and reachability analysis techniques are presented to elucidate and validate the performance of our proposed mobility model. The state space methods allow us to algorithmically derive the system behavior and rectify the errors of our proposed model.

## Introduction

Wireless networking has received unprecedented attention from the research community during the last decade due to the fast growth in technology. It has witnessed extensive development and created new horizons of communication. Wireless communication networks have several types, and one example is wireless mesh networks (WMNs) [1]. The mobile hosts (MHs) or simple stations (STAs) in wireless communications have dynamic nature and move in terrain according to different mobility profiles. Mobility models are used to mimic the traveling patterns of mobile hosts. Therefore, an underlying mobility model that dedicates the movement behavior to MH has a direct influence on the performance of these networks. This is due to their characteristics such as dynamic topology, multi-hop communication, security, shared media, entropy measure and eigenvalue measure in complex systems.

### 1.1 Mobility and Mobility Models in Wireless Networks

Mobility models have two broad categories and those are trace based and synthetic mobility models. In general, traces are accurate realistic mobility patterns that are derived from real life scenarios, particularly where a large number of participants are involved. However, new network environments like mobile ad hoc networks (MANETs) or WMNs cannot be easily modeled because they have not been deployed in large scale yet. Hence, researchers proposed various synthetic models without traces where the objective was to capture the characteristics of mobile nodes (MNs) without using traces. Synthetic mobility models attempt to model the mobility of each mobile host (MH) in a simplistic manner based on direction, speed, transition time between home and destination, the length between two points, and distribution of MH positions. A comprehensive study of these synthetic models can be found in [2][3].

The major role of mobility model is to generate the movement patterns according to real life scenarios in simulation based studies. An individual mobility model can be applied into a wide range of systems particularly the dynamics in mobile scenarios are of great interest [4][5]. Moreover, the authenticity of a mobility model depends on how it produces more realistic mobility patterns. A precise and realistic mobility model generates more accurate simulation results to evaluate network parameters. Researchers have developed numerous mobility models offering diverse characteristic in simulation based research. Some models are simplistic and others are based on comprehensive modeling approach to generate realistic travelling patterns. Mobility models can be divided into seven broad categories: (i) individual mobility models, (ii) group mobility models, (iii) flocking mobility, (iv) auto-regressive mobility models, (v) non-recurrent mobility, (vi) virtual game-driven mobility, and (vii) time-variant community mobility [6]. The accuracy of the mobility model is highly dependent on the degree of realism. Some other mobility models are customized for specific scenarios. The most popular random mobility models are random walk mobility model [7] and its variations, random waypoint mobility model (RWP) [8], and random direction mobility model [9]. In recent years, several studies have been published with consensus that existing mobility models such as the well-known random waypoint (RWP) are insufficient to represent realistic mobility patterns. Remarkable effort was made in [10] to compare the evaluation methods and it concluded that 75.5% research is based on simulations and majority of papers (73.8%) implemented well known RWP mobility model. In addition to its wide applicability and popularity, there is broad criticism in numerous studies (e.g., [6][11][12][13][14][15][16][17][18]) on RWP and other random models. The key issues are sudden stops, speed decay, density wave issue, steady state problem, and the so-called border effect phenomena. Moreover, the mobility model does not retain any preceding information; therefore, the memory-less decisions for the next itinerary may generate unrealistic movement patterns. Finally, there is an increasing consensus among the network research community that the existing mobility models are either unrealistic or tailor-made for specific situations, and this causes the degradation of quality of service (QoS) for wireless networks.

To alleviate the above issues, this paper presents a random SHER mobility model based on movement patterns observed from a social and behavioral network for a perspective user. The objective is to enhance the model such that it can monitor the behavior and position of the MHs on a regular basis. If the network has enough information about the MHs attraction points or hotspots, then the model can implement intelligent strategies to accurately predict the MH’s current or future location based on previous observations. Consequently, the network can reserve its resources for appropriate time by sending minimum location finding messages.

Traditionally, simulation is a method to conduct wireless networks research because of its numerous benefits. However, the study of literature reflects that newcomer students and researchers often get perplexed in the complexity of simulators and lose their concentration [19]. Formal methods [20] are mathematical-based techniques that have been proven as an appropriate tool for investigation and rectification of the model’s attribution and rectification. This approach is typically motivated because of its power to detect inconsistency and reduce the “time to market”. Conventional methods have rigorous computer based tool support, which ticks off all possible execution paths throughout the system and reveals virulent concurrent errors that have not been unveiled by implementing other testing strategies. Graphs have also been used as a formal approach and to describe the characteristics and entropy-based measures of complex networks, researchers investigated many graph invariants, e.g. [21][22][23][24].

Colored Petri nets (CPNs) [25][26] are bipartite graphs suitable for development of concurrent discrete events and distributed systems. Many techniques such as timed color token, hierarchical modeling, capacity bounding, priority queues, recursive functions, synchronous, and asynchronous behaviors are used to model these complex systems. CP-nets combine Petri nets [27] and functional programming language Standard ML (SML) [28] to provide a concrete platform to researchers. CPN Tools [29] is a powerful software for modeling and analysis of the CPN models, and is widely accepted and acknowledged by the research community.

The movement patterns of the mobility model play a vital role in the performance evaluation of wireless networks. The literature demonstrates that very few publications can be found that address the mobility issues in a wireless network using CPNs. A magnificent work of Kristensen et al. [30] can be considered as a landmark in CPN based modeling of the network protocols. The authors abstractly described both micro and macro mobility architecture scenarios in ad-hoc networks. Their focus was to develop a model that integrates conventional protocols with ad-hoc network protocols. A valuable contribution of their work is that the CPN model reflects communication and mobility functionality in a single model. The model is appropriate for mobility test cases. The work of Xiong et al. [31] has a profound influence on the mobility in ad-hoc networks. To overcome the dynamically changing topology problem, they proposed a topology approximation (TA) architecture using CPNs. They implemented their proposed mechanism to build a CPN model based on an Ad Hoc on Demand Distance Vector (AODV) routing protocol. Their results showed that TA mechanism exhibits realistic movement characteristics and is able to mimic the dynamic changes of the network topology.

Capturing mobility through CPN is the most challenging activity. Khan et al. [32] presented a CPNs based formal model in the well-known random walk mobility model for WMN without border effect and speed decay problems. The proposed model can be easily modified to generate complex mobility patterns. An elegant CPN based synthetic RWP mobility model was presented in [33]. Despite their contributions, the main limitation is that the proposed model was not able to generate the trajectory of every epoch. In order to produce realistic movement patterns, we implemented RWP mobility model in [34] by removing the border effect and speed decay issues. The model was able to generate different mobility patterns based on the diverse topology. Those patterns can further be used to address the mobility management issues in wireless communication networks. A nice effort was presented in [14] and the authors presented a group mobility model that considers the psychological and sociological behavior of the mobile nodes. The model was able to capture the natural behavior as fork-join and generated the realistic motion of the living creatures. To produce several mobility patterns, the authors in [35] presented a generalized random mobility model including hotspots over one-dimensional terrains. The model had the functionally to capture points that the user had higher probability to spend time. Recently, we introduced an accurate and extendable abstract level hierarchical CPN based formal model of a community based wireless mesh network where a user can move or send data packets [36]. The aim of the proposed CPN model was to elaborate the communication mobility requirements, and this was the first step of the systematic verification in the WMN environment at an abstract level.

Although some of the above efforts were made to model realistic behaviors of the mobility models, no one attempted to cover diverse aspects of the MHs movement compared to the SHER mobility model. A significant difference between SHER and other random or deterministic mobility models is the simplicity and generality. Most of the existing models are implemented in simulators and their underlying mathematical models are complex. On the contrary, our implementation is based on the formal modeling and a new module can easily be added or existing modules can be modified to produce more complex scenarios such as obstacles in the mobility of the nodes.

### 1.2 Research Contributions

The significant contributions of the work presented in this paper can be summarized as follows:

- Temporal dependency of velocity: To select the next epoch parameters and provide steady state, SHER mobility model considers the previous velocity and gradually increase, decrease or remain constant for the next epoch’s speed.
- Temporal dependency of destination: To select the next itinerary, SHER mobility model considers previous information; hence, MH’s new destination is intelligently dependent on its moving history.
- Sudden stops: The proposed approach provides complete removal of the abrupt stops for MHs.
- Speed decay problem: Our implementation does not exhibit speed decay issue, and the MH’s speed remains constant during each trip using the proposed application.
- Pause time dependency: Random pause time after completion of each trip is dependent on the destination location. For example, the pause time for location “A” is more than location “B” and “C” in a priority based method. This type of pause time reflects a more realistic attitude of the mobile user and it is employed in the SHER model.
- Border effect problem: Border effect issue demonstrates that when an MH touches the boundary of terrain, then it bounces to the middle and concentrates. The SHER mobility model avoids this type of behavior.
- Spatial dependency: Spatial dependent behavior of the nodes influenced by previous information can be monitored using our implementation.
- Uniform nodal distribution: On start and during execution, the SHER maintains uniform distribution.
- Mini simulator: The SHER mobility model can be executed in CPN Tools’ user friendly environment that is freely available [29]. The SHER mobility model, in an executable *SHER*.*cpn* file, enables the user to generate numerous different mobility patterns using a single mouse-click, even if a researcher is not much familiar with formal methods or CPNs.

To demonstrate the impact of this model, we validate its functional properties using extensive simulations and state space analysis techniques. The proposed model will provide an encouragement to researchers for the specification, modeling, and verification of communication protocols using CPNs to avoid the complexities of simulators. The rest of the paper is divided into four main sections. Section 2 concentrates the formal modeling of the SHER mobility model using CPN tools. Section 3 examines the state space of the models and verifies the results. We summarized our achievements in Section 4 where we also present our conclusions and future directions.

## CPN Based Modeling for the SHER Mobility Model

### 2.1 Environment Scenario

The SHER mobility model is developed using Petri net based formalism while basic concepts and terminology about Petri nets, its formal definitions, and CPN can be found in [37]. Moreover, it is a cumbersome task to create intricate models; however, similar to the other modular programming languages, CPN supports a hierarchical module concept in which large nets can be broken into smaller components.

**Fig 1:**
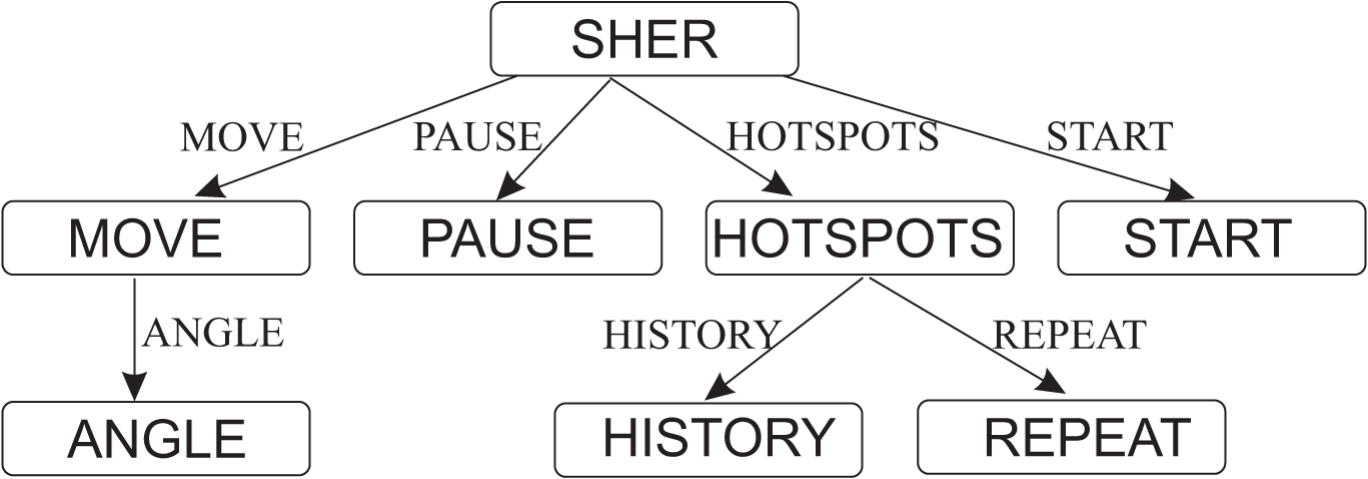
Instance hierarchy for the SHER mobility model.

The basic theme of the SHER synthetic random mobility model is based on the theory of the well-known RWP mobility model [8], which has already been implemented in many simulators such as NS-2 [38]. The CPN model of the SHER mobility model is organized as a set of eight modules. Because the system components are often used repeatedly, it is inefficient to draw these components from scratch every time. Fig 1 shows the graphical representation of the module *hierarchy* for the SHER model. The modules names are written inside the nodes. The arcs annotate the name of the corresponding substituting transitions.

The SHER module has no incoming arc; hence, it is the root of the hierarchy and is called the prime module. The SHER model consists of four main parts. Page MOVE with one sub-page ANGLE models the movement scenarios of an MH. Page HOTSPOTS is further decomposed into two subpages, HISTORY and REPEAT, and is responsible to keep track of every epoch of an MH and models the next itinerary based on the previous movement.

**Fig 2:**
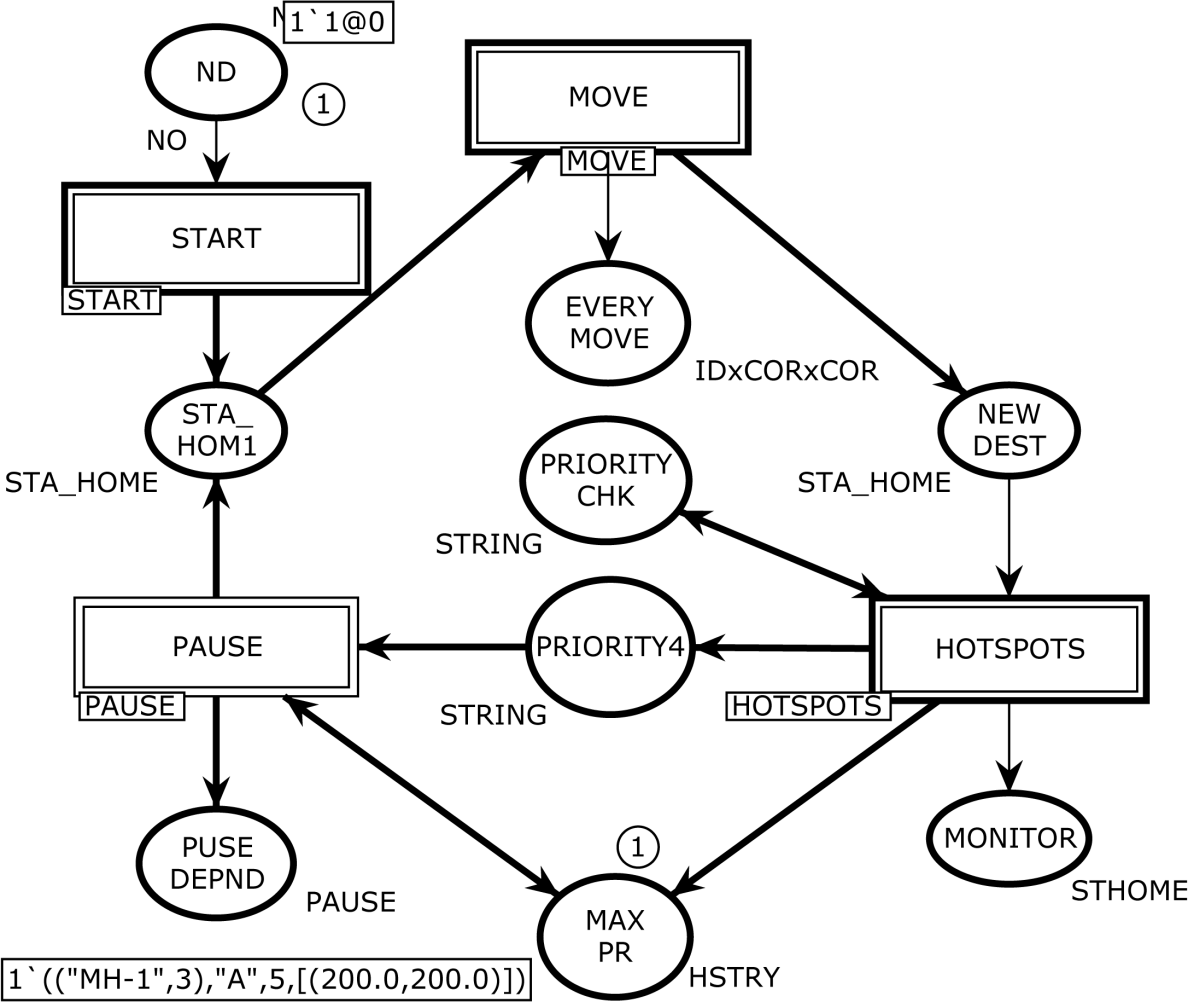
The SHER page–Architecture of the CPN model with initial marking *M*_*0*_.

In addition, the PAUSE page models the pause time dependency based on the attraction point’s category of the MH and finally the START module randomly selects the first trajectory movement coordinates and other parameters of an MH. The model has a dynamic nature; hence, an MH can be placed in *100 × 100 m* ^*2*^ communication area using a uniform random distribution. The model contains a finite set of places *P*, a finite set of transitions *T*, a set of directed arcs *A*, a finite set of non-empty color sets *∑*, a finite set of typed variables *V*, a color set function *C* that assigns a color set to each place, a guard function *G*, an arc expression function *E* that assigns an arc expression to each place, and an initialization function *I*. Fig 2 depicts the abstract SHER module that has four *substituting transitions* MOVE, START, PAUSE, and HOTSPOTS as indicated by the associated HS-tag and nine places, which are ND, STA_HOM1, PRIORITY CHK, EVERY MOVE, NEW DEST, PRIORITY4, MAX PR, PAUSE DEPND, and MONITOR.

The declarations of the color sets used in the SHER module (see Fig 2) are listed in Table 1. The color sets are defined using the basic Standard ML types. A constant tn, which represents the total nodes, is *globally* declared as one and it has a type Val. The color set NO is used for identity (ID) number of an MH with the form MH-(i) where 1 ≤ i ≤ tn. The color set RCOOR (Real Coordinates) is defined as a *product color set* of the simple color set RCOR and LCOOR. In addition, the color set RCOOR is of a type *list*.

**Table 1:** Declaration of the color sets for the SHER page.

Line No.	Color Name	Color Type
1.	colset NO	int with 1..tn timed;
2.	colset INT	int;
3.	colset ART	int;
4.	colset REAL	real;
5.	colset STRING	string;
6.	colset ID	string;
7.	colset RCOR	REAL;
8.	colset RCOOR	product RCOR*RCOR;
9.	colset LCOOR	list RCOOR;
10.	colset NODE	product ID*ART timed;
11.	colset STA_HOME	product NODE*LCOOR*LCOOR timed;
12.	colset PAUSE	product INT*NODE*STRING*LCOOR timed;
13.	colset STHOME	product INT*NODE*LCOOR*STRING*LCOOR timed
14.	colset IDxCORxCOR	product INT*NODE*LCOOR*LCOOR;
15.	colset HSTRY	product NODE*STRING*INT*LCOOR;

The color set NODE denotes the MH ID number with its arrival time. The color set STA_HOME is used to model the node ID with home and destination coordinates for the next epoch. The place MONITOR has a color set STHOME whose elements are 5-tuples consisting of sequence number, node, home coordinates, hotspot, and destination. The color set PAUSE is used to model the pause time after completing every epoch based on the attraction point category. The place EVERYMOVE with color set IDxCORxCOR is used to keep track of the every move of an MH. The places PRIORITYCHK and PRIORITY4 with color set STRING are used in modeling the attraction point category. The place MAX PR has color set HSTRY whose elements are 4-tuples and shows top attraction points of n MH.

### 2.2 Hierarchical Pages of the CPN Model

**Fig 3:**
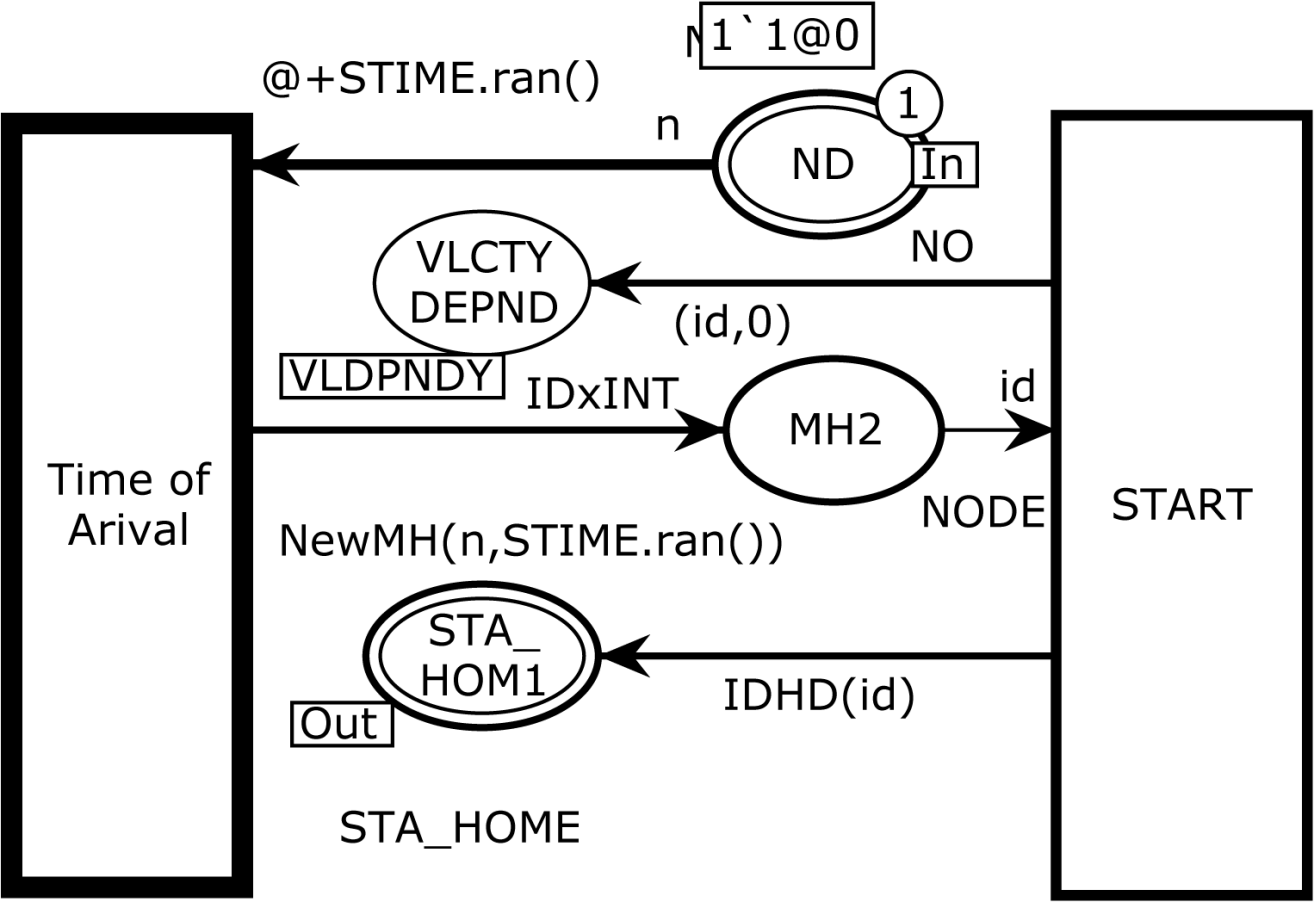
The START module with initial marking *M*_*0*_.

The Hierarchical START page is depicted in Fig 3 and is the sub-module of the *substituting* transition START in Fig 2. The functionality of this page is to produce an MH for simulation. This module contains four *places* and two ordinary *transitions*. The transition TimeofArival models the mobile node identity and its arrival time. The variable n is of type NO, and the variable id is of type NODE (see Table 1). In initial marking *M0*, this transition is enabled and after one token is fired, it moves to the place MH2 of color set NODE. The place VLCTYDEPND is a *fusion* place and is a member of *fusion* set VLDPNDY. A *fusion* set allows different places from multiple pages to be glued together and all member places always share the same tokens.

The place ND in this module is called port place and represents the total number of MHs. Place ND is an *input socket port* $P_{sock}^{in}\;(t)$ while place STA_HOM1 is an *output socket port* $P_{sock}^{out}\;(t)$.

$$P_{sock}^{in}\;(START)=\{\text{ND}\}$$

$$P_{sock}^{out}\;(START)=\{\text{STA}\_\text{HOM}1\}$$

$$VLDPNDY={\text{VLCTY}\;\text{DEPND}}^{START},{VLCTY\;DEPND1}^{ANGLE}\}$$

P={MH2}

T={START,Time of Arival}

After firing transition TimeofArival, the following two-tuple token are moved to the place MH2, which has NODE color set. The function *New MH* takes two inputs of the host ID and arrival time and produces the following token of type NODE:

MH→1`("MH-1",3)@3

**Fig 4:**
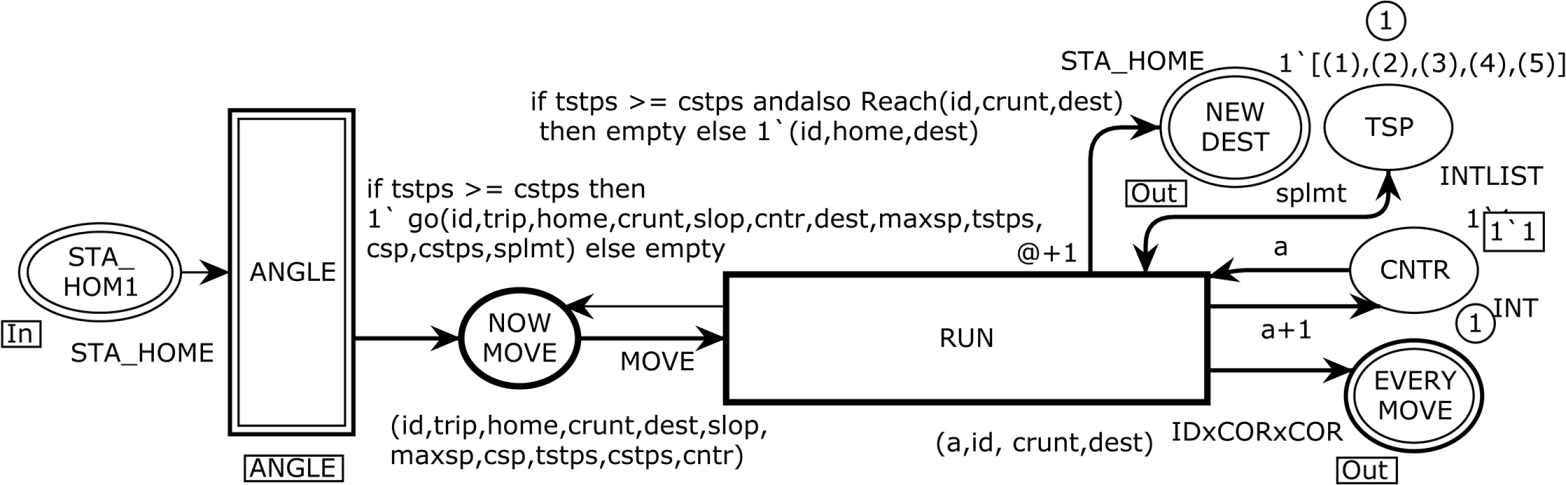
The MOVE module models the mobility management.

Fig 4 illustrates page MOVE, which is the sub module of the *substituting* transition MOVE in Fig 2 and is responsible for mobility management of an MH. This page has two ordinary places, two *output socket* places $P_{sock}^{out}\;(t)$, one *input socket* plac*e* $P_{sock}^{in}\;(t),$ and two *transitions*, one ordinary and one substituting as noted below:

$$P_{sock}^{out}(MOVE)=\{\text{NEW DEST},\;\text{EVERY MOVE}\}$$

$$P_{sock}^{in}(MOVE)=\{\text{STA}\_\text{HOM}1\}$$

$$T_{sub}={\text{ANGLE}}^{MOVE}$$

P={TSP,CNTR}

T={RUN}

fun Reach (id:NODE,x1:LCOOR,x2:LCOOR)

This function is used to monitor whether MH has reached the destination or not. The Reach function takes a three-tuple as domain and returns a type boolean.

fun slow(csp:INT,maxsp:INT,tstps:INT,cstps:INT,splmt:INTLIST)

This function is used to model the removal of sudden stops problem. To exhibit realism, MH starts with low speed and gradually increases up to the maximum speed limit, and gradually decreases when it reaches the destination.

fun xaxis (x:LCOOR, a:REAL, c:REAL)

This function generates the next x-axis according to the parametric equation formula. *x = r cos(t)*

fun yaxis (x:LCOOR, a:REAL, c:REAL)

This function generates the next y-axis according to the parametric equation formula. *y = r sin(t)*

fun go(id:NODE,trip:INT,home:LCOOR,crunt:LCOOR,slop:RCOR,

cntr:REAL,dest:LCOOR,maxsp:INT,tstps:INT,csp:INT, cstps:INT,

splmt:INTLIST)

The go function is used to restrict the MH to move to the next coordinate after satisfying all imposed conditions.

The sub module ANGLE computes the desired angle and other information to reach its destination and transmits eleven-tuple token to the NOWMOVE place. The RUN transition in Fig 4 is the most important one in the model, and has three *input* arcs and five *output* arcs. It models the actual movement of an MH according to a specified criteria. We define two functions Reach and go for the use in arc expression from transition RUN to NOWMOVE place. The go function calls three functions: slow, xaxis, and yaxis. These functions are described as follows:

When RUN transition occurs, it fetches one token from the NOW MOVE input place with input arc expression (id,trip,home,crunt,dest,slop,maxsp,csp,tstps,cstps,cntr), one token from the CNTR place, and one token from the TSP place. The variables of the incoming arc expressions are declared as:

var slop: RCOR; (* The itinerary angle of movement*)

var trip,maxsp,csp,cstps,: INT; (* The integers are used to describe trip number, *maximum*

tstps,cntr,a *speed limit*, *current speed*, *current step number*, *total steps of epoch*, increasing counter *)

var home,crunt,dest: LCOOR; (* *The home*, current and destination coordinates of MH*)

var id: NODE; (* *The Identity Number of MH*, with time*)

var splmt: INTLIST; (* The current status of MH*)

The following arc expression is implemented on an outgoing arc from the transition RUN to the NOW MOVE place.

if tstps > = cstps andalso Reach(id,crunt,dest) then 1`go(id,trip,home,crunt,slop,cntr,dest,maxsp,tstps,csp,cstps,splmt) else empty

If the above condition is satisfied, then eleven-tuple token will be moved back to the NOW MOVE place after increasing one step to the next coordinates.

**Fig 5:**
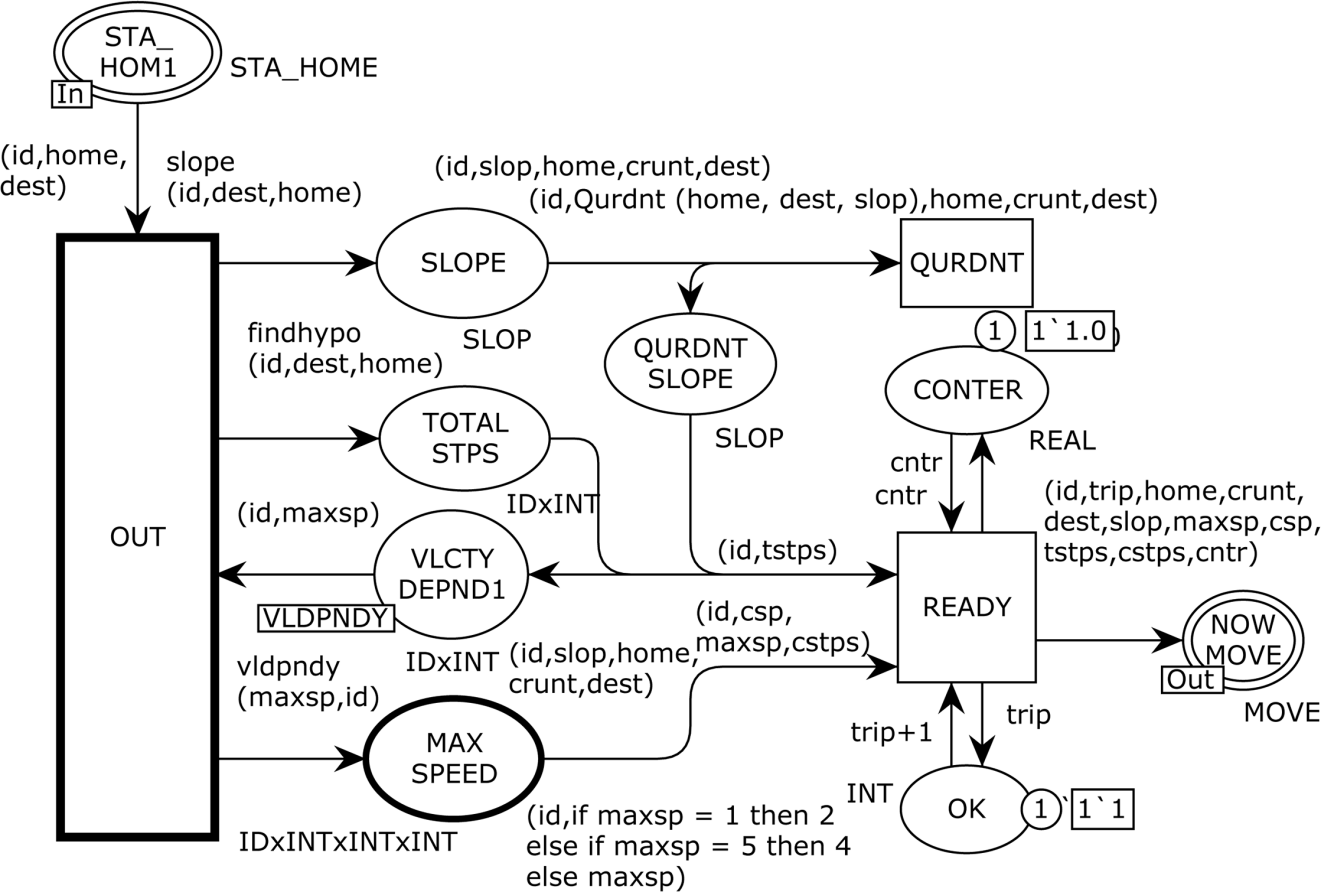
The ANGLE module which is responsible to demonstrate the moving paramaters.

The substituting transition ANGLE in Fig 3 corresponds to the modeling of moving parameters and changes for every epoch. Fig 5 shows the ANGLE module, which enables the CPN to model the velocity dependency, angle to move, and total number of steps to reach the destination. This page has seven ordinary places, one *input socket* place, i.e., $P_{sock}^{in}\;(t)$, one *output socket* place, i.e. $P_{sock}^{out}\;(t),$ and two ordinary transitions as presented below:

$$P_{sock}^{in}(ANGLE)=\{\text{STA}\_\text{HOME}\}$$

$$P_{sock}^{out}(ANGLE)=\{\text{NOWMOVE}\}$$

P={SLOPE,QURDNT,CONTER,QURDNTSLOPE,TOTALSTPS,OK,VLCTYDEPND1,MAXSPEED}

T={OUT,READY}

The output arc from the OUT transition applies three functions, i.e., slope with product (id,dest,home), findhype with product (id,dest,home), and vidpndy with product (maxsp,id). When the OUT transition occurs with a given binding, it fetches one token from the STA_HOME1 and VLCTYDEPND1 places to evaluate the arc expressions of the output arcs to the SLOP, TOTALSTPS, and MAXSPEED places. The slope function takes the expression (id,dest,home) as its arguments and returns the angle to move towards the destination based on the current coordinates using the CPN tools built-in *Math*.*atan()* function. The findhype function with the expression (id,dest,home) as its argument is used on the arc from the OUT transition to the TOTAL STPS place to calculate the total steps from home to destination by implementing the *Math*.*sqrt ()* and *Math*.*pow()* functions. The vidpndy function with the arguments (maxsp,id) is used to derive the mobility characteristic, which is the *temporal dependency of velocity* to represent a more realistic behavior by incorporating the velocity dependency of an MH. This function calls the *VLCTY*.*ran()* function to decide the next epoch speed depending on the previous speed to avid the sudden increase or decrees in speed. Now we consider the QURDNT transition, which implements Qurdnt function on outgoing arc towards the QURDNT SLOPE place to find quadrants of the destination to finalize the moving angle. An MH can be in any one of four quadrants, i.e., Quadrant-I (0^o^ - 90^o^), Quadrant-II (90^o^ - 180^o^), Quadrant-III (180^o^ - 270^o^), and Quadrant-IV (270^o^ - 0^o^). Then, the READY transition with eleven-tuple arc expression to the port NOW MOVE place produces final input to the MOVE module (see Fig 4) for traveling the MH to the destination. The READY transition fetches five tokens from the input places of CONTER, OK, QURDNT SLOPE, TOTAL STPS, and MAX SPEED and produces four tokens to the output places of CONTER, OK, and VLCTY DEPND1. The CNTR (counter) and OK places are used to model the incremental counters to keep track of every epoch sequence.

**Fig 6:**
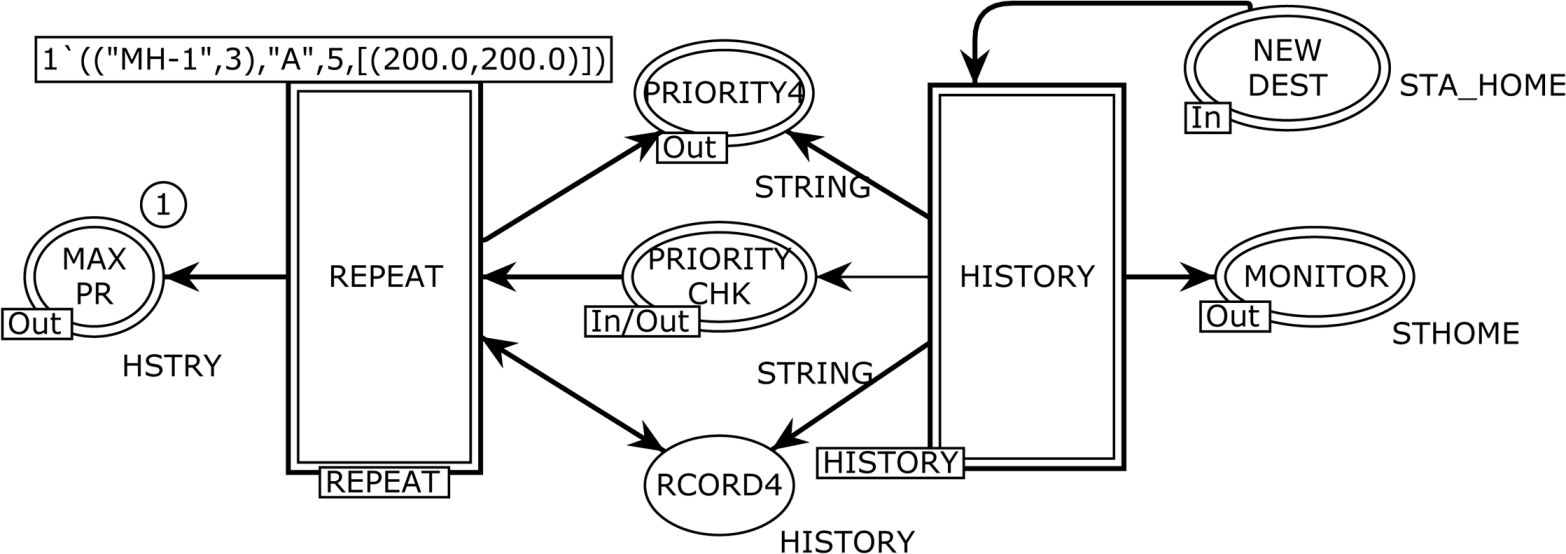
The HOTSPOTS module to resolve the memory-less issue.

Fig 6 depicts the abstract HOTSPOTS page, which renders the *temporal dependency* behavior to overcome the memory-less nature problem of the RWP and its other variants. The HOTSPOTS module corresponds to the *substituting* transition HOTSPOTS in Fig 2, and it is used to tie the REPEAT and HISTORY sub-modules. This page has one ordinary place, three *output socket* places $P_{sock}^{out}\;(t)$, one *input/output socket* plac*e* $P_{sock}^{i/o}\;(t),$ one *input socket* plac*e* $P_{sock}^{in}\;(t)$, and two substituting transitions as presented below:

$$P_{sock}^{in}(HOTSPOTS)=\{\text{NEW DEST}\}$$

$$P_{sock}^{out}(HOTSPOTS)=\{\text{MAX PR},\text{PRIORITY}4,\;\text{MONITOR}\}T_{sub}={\text{HISTORY}}^{HOTSPOTS},{\text{REPEAT}}^{HOTSPOTS}$$

$$P_{sock}^{\frac{i}{o}}(HOTSPOTS)=\{\text{PRIORITY CHK}\}$$

P={RCORD4}

The five input, output, and input/output places constitute an interface through which the HOTSPOTS module exchanges tokens with other pages. The declarations of the color sets used in the HOTSHOTS module and its two sub modules (see Fig 6) are listed in Table 2. The process to find attraction points (hotspots) is modeled using the HISTORY and REPEAT substituting transitions.

**Table 2:** Declaration of Color set for HOTSPOTS and its substitution transitions.

Line No.	Color Name	Color Type
1.	colset STHOME	product INT*NODE*LCOOR*STRING*LCOOR timed;
2.	colset HISTORY	list HSTRY;
3.	colset IDxSTxCOR	product NODE*STRING*LCOOR;
4.	colset HSTCNTR	product NODE*INT*INT*INT*STRING*LCOOR;
5.	colset IDxINTxINTxINT	product NODE*INT*INT*INT;
6.	colset TPLACE	list IDxSTxCOR;

The HISTORY substituting transition in Fig 6 corresponds to the modeling of the HISTORY module, which is used to keep the track record of attraction points of the MH as shown in Fig 7. The HCNTR, PRITY PLACES, and PHIST places are *fusion* places. There are nine ordinary places, one *input socket* place $P_{sock}^{in}\;(t)$, four *output socket* places $P_{sock}^{out}\;(t),$ and five ordinary transitions as listed below:

$$P_{sock}^{in}(HISTORY)=\{\text{NEW DEST}\}$$

$$P_{sock}^{out}(HISTORY)=\{\text{MONITOR},\text{PRIORITY CHK},\;\text{PRIORITY}4,\text{RCORD}4\}$$

$$HCnters=\{{\text{HCNTR}}^{HISTORY},{\text{HCNTR}3}^{PAUSE}\}$$

$$TPRITY={\text{PRITY PLACES}}^{HISTORY},{\text{PRITY PLACES}2}^{PAUSE}\}$$

$$PrityChk={\text{PHIST}}^{HISTORY},{\text{PHIST}2}^{PAUSE}\}$$

P={PHIST5,PRIORTY2,HCNTR4,PHIST9,PHIST8,PRIORITY,HSTY3,RCORD3,HCNTR2}

T={PRITY,H3ND,H2ND,UPHSTRY,CHKHIST}

**Fig 7:**
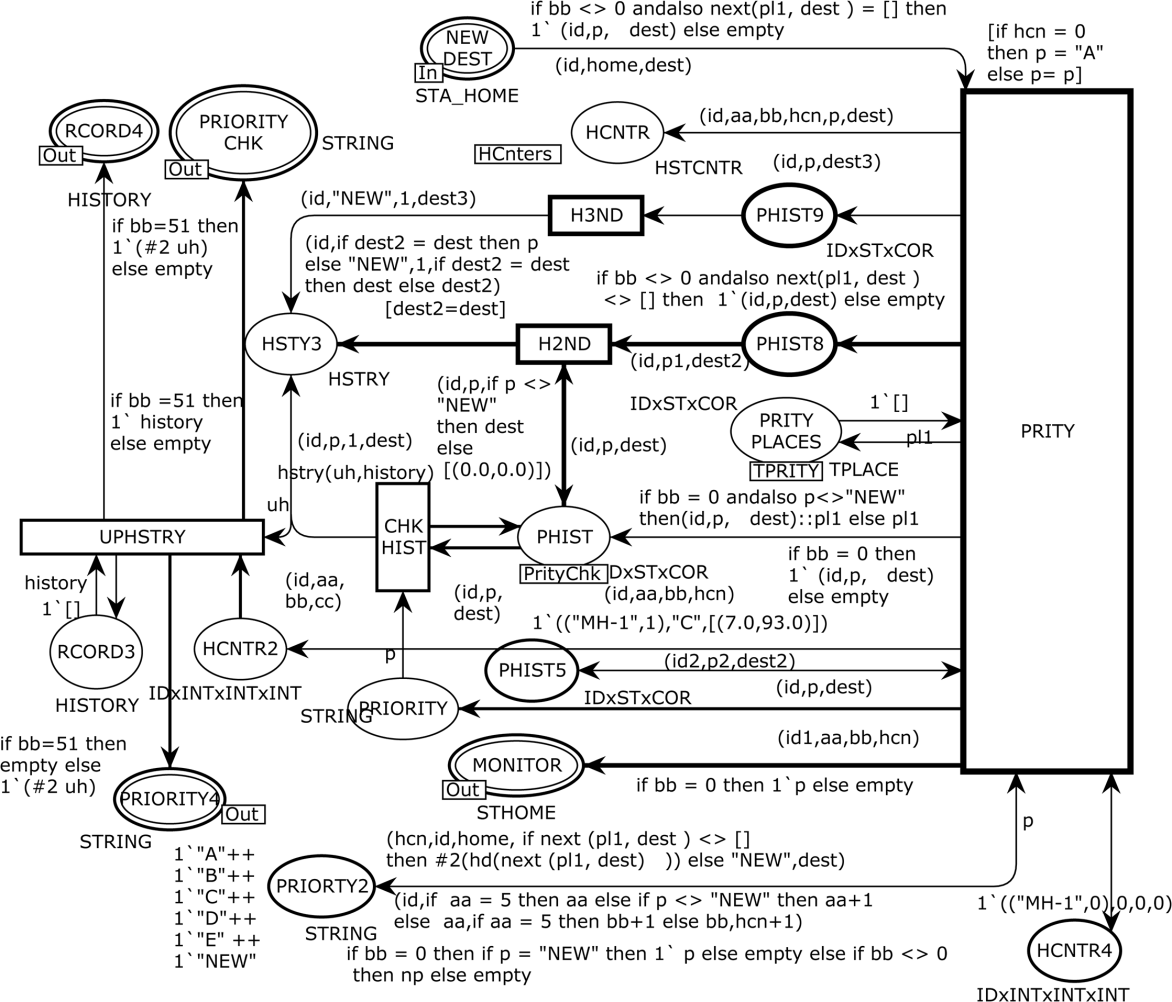
The HISTORY module which models the attraction points of MH.

The PRITY transition fetches five tokens from the input places of NEW DEST, PHIST5, PRIORITY2, PRITY PLACES, and HCNTR4 and produces tokens to the output places of HCNTR, PHIST5, PRIORITY2, HCNTR4, MONITOR, PRITY PLACES, PHIST, PHIST8, PHIST9, HCNTR2, and PRIORITY.

We define a polymorphic function next to use as an arc expressions from the PRITY transition to the MONITOR and PHIST8 places with a product argument (pl1, dest). When the PRITY transition occurs with a given binding, it yields one token to the HCNTR4 place to keep track of three counters, one token to the output PRITY PLACES place to maintain the history of attraction points of the mobile user, and one five-tuple token to the MONITOR place for monitoring and statistical analysis requirements. The places HCNTR, PRIORITY, PHIST9, HSTY3, PHIST, and PHIST8 assist places used in developing the CPN model to extract the history based information. The CHKHIST transition models a four-tuple arc expression (("MH-1",0),"A",1,[(40.0,97.0)]) for the HISTY3 place where the first tuple reflects an MH ID, the second element represents the attraction point, the third one is a counter, and the fourth represents the coordinates. Then, we implement three arc expressions on the input and four on the output arcs of the UPHSTRY transition. The recursive function hstry takes expression (uh,history) as arguments and updates the list of attraction points. It obtains an attraction point p from the variable uh to be added and a list history, as parameters. Initially, the list is empty, and we add attraction point p to the list. In the inductive step, we first check whether the head of the list and attraction point p have the same attraction point attribute. If this is the case, then we increment the third tuple priority counter by one to show the total number of trips to the specific destination. If the attraction points are different, then we add the attraction point p with its coordinates to the next element on the history list. This is done using the hstry function to the variable uh and tail of the history list.

**Fig 8:**
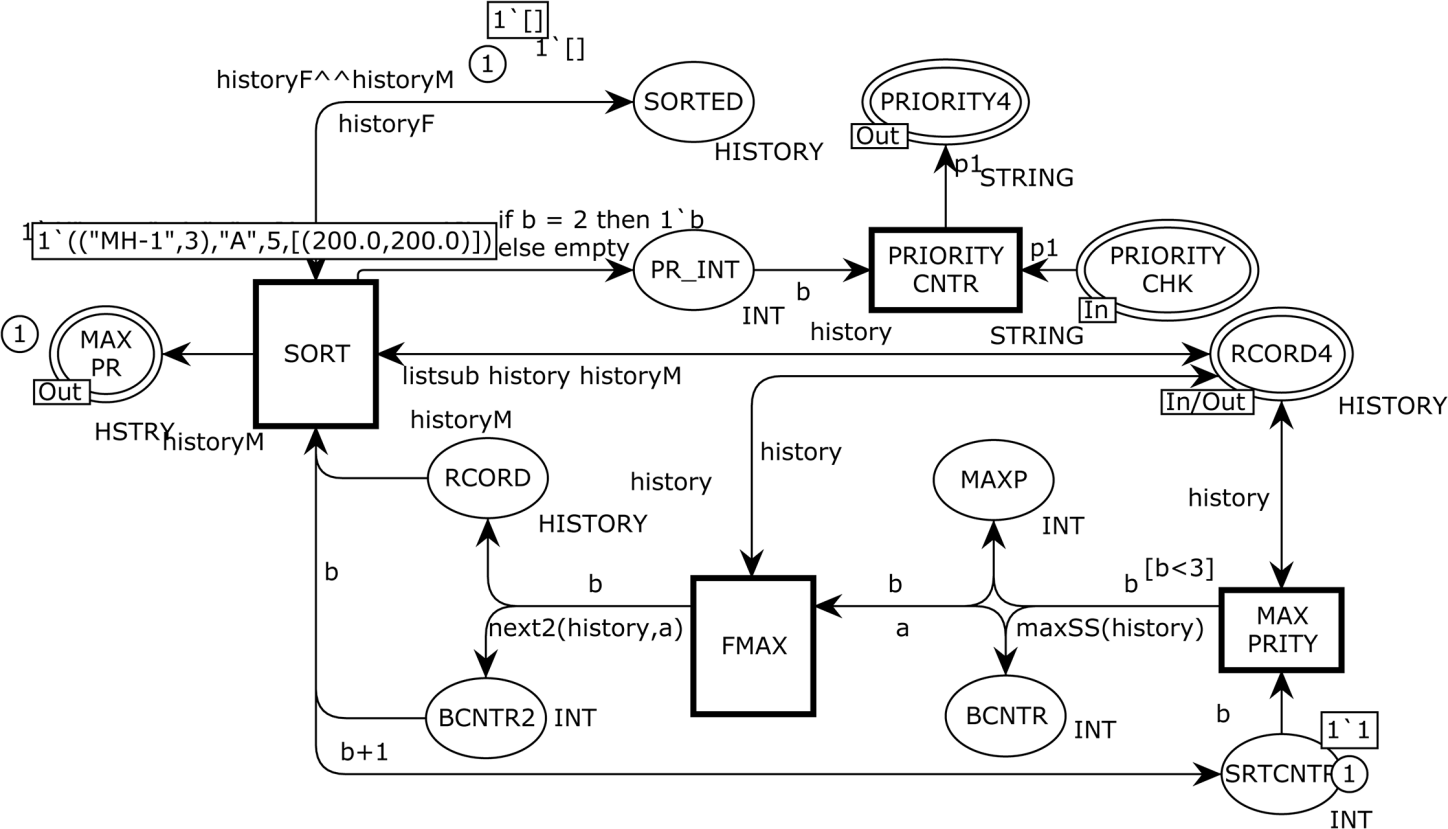
The REPEAT module which represents final five attraction points based on frequency of visits.

The instance reflected in Fig 8 corresponds to the REPEAT substituting transition in Fig 6, which is used to model the final sorted list of attraction points based on the maximum number of trips of the MH. There are seven ordinary places, one *input socket* place $P_{sock}^{in}\;(t)$, one *input/output socket* plac*e* $P_{sock}^{\frac{i}{o}}\;(t),$ two *output socket* places $P_{sock}^{out}\;(t),$ and four ordinary transitions as follows.

$$P_{sock}^{in}(REPEAT)=\{\text{PRIORITY}\}$$

$$P_{sock}^{out}(REPEAT)=\{\text{MAX PR},\text{PRIORITY}4\}$$

$$P_{sock}^{i/o}(REPEAT)=\{\text{PRIORITY CHK}\}$$

P={SRTCNTR,BCNTR,MAXP,SORTED,PR_INT,RCORD,BCNTR2}

T={MAX PRITY,SORT,FMAX,PRIORITY CNTR}

The REPEAT module of the SHER mobility model enables only once when the integer counter, i.e., <bb = 51> is evaluated by the UPHSTRY transition via an arc expression to the output RCORD4 place (see Fig 7). We specified five types of attraction points as *“A”*, *“B”*, *“C”*, *“D”*, *and “E”*. Other type of an attraction point is *“NEW”* and specifies that the MH traveled to a new destination other than the five declared attraction points. The REPEAT module finds two *hotspots* from the five attraction points and identifies these as the *hom*e and *work place* of an MH, where it repeatedly travels. The maximum frequency of trips (the third attribute of the history variable) is declared as hotspot *“A”* and the second highest is *“B”*. The MAX PRITY transition takes a list of attraction points with their visiting frequency as an input from the port RECORD4 place and generates the first hotspot based on the maximum number of visits using the maxSS (history) function to the output MAXP place. The SRTCNTR place with INT color set maintains a counter to the MAX PRITY bound transition to produce two hotspots. The maxSS() recursive function takes the expression (history) as an argument and produces an integer showing the maximum number of visits. This integer is further used by the FMAX transition as an input to determine the first hotspot. The FMAX transition fetches three tokens from the MAXP, BCNTR, and RCORD4 places and its objective is to produce the first hotspot using the next2(history,a) function to the output RECORD place. The next2() function initially takes an empty list, then we add the maximum visits using the variable a from the MAXP place. Its other input is from the RECORD4 place, which contains a complete record of attraction points. In the next step, we check whether the head (3^rd^ attribute) of the list and variable a are equal. If this is the case, then the next2() function produces a *head* to the RECORD place; otherwise, this function is implemented to the *tail* of the history list. Now we consider the transition SORT, which delivers the first hotspot to the MAX PR and SORTED places. This transition has four input and five output places with expressions. After finding the first hotspot the same process is repeated for the second hotspot, and the expression <b + 1> to the output arc of the SRTCNTR place enables the MAX PRITY transition to produce the next hotspot. An occurrence of the PRIORITY CNTR transition models the control transfer to the PAUSE module and is enabled once during the simulation with bindings <b = 2> and <bb = 51>.

**Fig 9:**
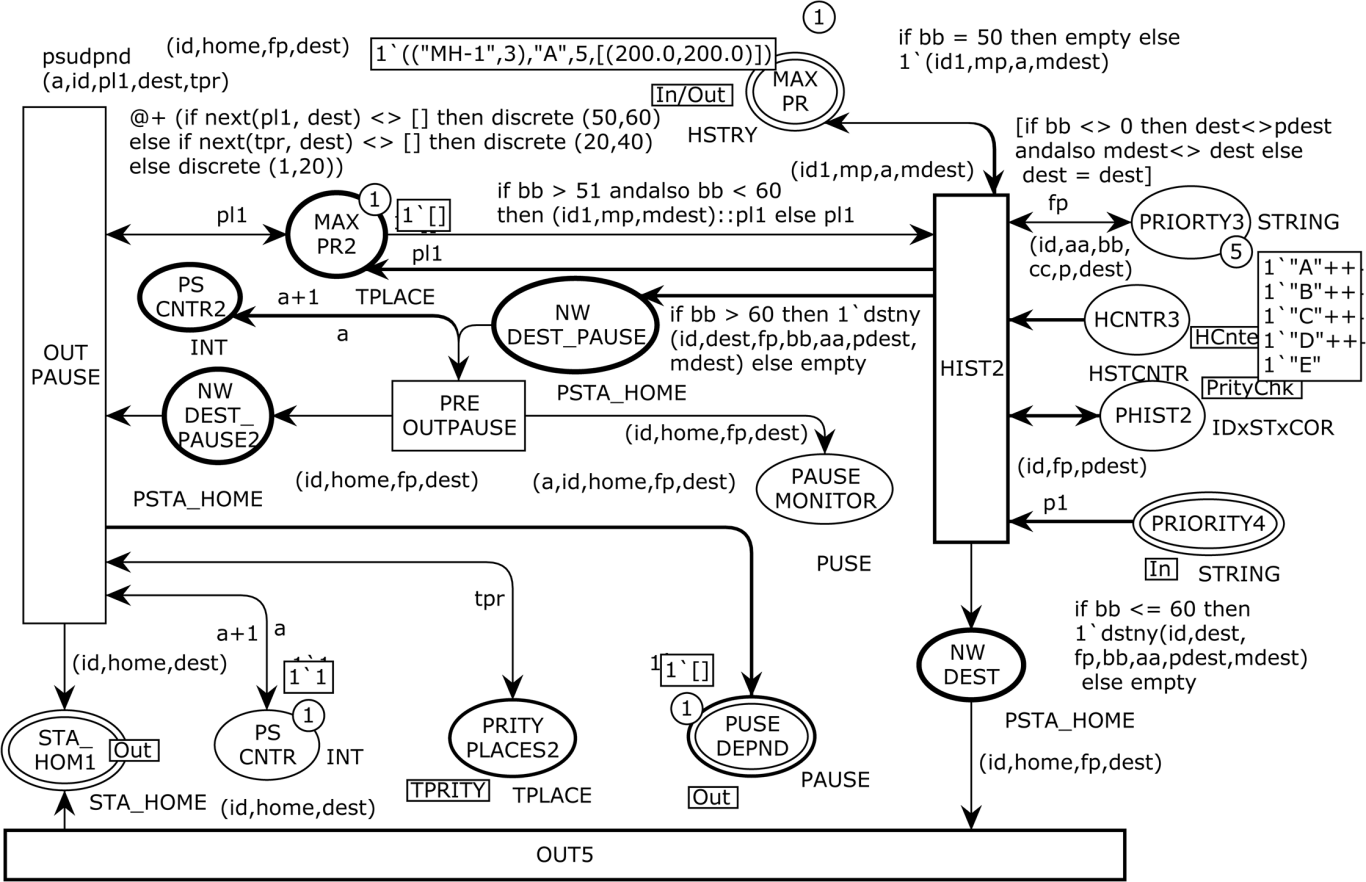
The PAUSE module to show the next destination based on previous history.

Fig 9 shows the PAUSE module. The PAUSE substituting transition in Fig 2 corresponds to this module and is used to decide the next destination based on the previous history. This sub-process also models the pause time dependency based on the hotspot’s category. In a real time scenario, a user spends more time in home or office; therefore, this realistic behavior is represented by this PAUSE module. Three places of PRITY PLACES2, HCNTR3, and PHIST2 are *fusion* places. There are eight ordinary places, one *input socket* place $P_{sock}^{in}\;(t)$, one *input/output socket* plac*e* $P_{sock}^{i/o}\;(t),$ two *output socket* places $P_{sock}^{out}\;(t)$, and four ordinary transitions as follows.

$$P_{sock}^{in}(PAUSE)=\{\text{PRIORITY}4\}$$

$$P_{sock}^{out}(PAUSE)=\{\text{STA}\_\text{HOM}1,\text{PAUSE DEPND}\}$$

$$HCnters={\text{HCNTR}}^{HISTORY},{\text{HCNTR}3}^{PAUSE}\}$$

$$TPRITY=\{{\text{PRITY PLACES}}^{HISTORY},{\text{PRITY PLACES}2}^{PAUSE}\}$$

$$PrityChk={\text{PHIST}}^{HISTORY},{\text{PHIST}2}^{PAUSE}\}$$

$$P_{sock}^{\frac{i}{o}}(PAUSE)=\{\text{MAX PR}\}$$

P={PRIORTY3,MAX PR2,PS CNTR,NEW DEST_PAUSE,NEW DEST,PS CNTR2,PAUSE MOINTOR}

T={HIST2,OUT PAUSE,OUT5,PRE OUTPAUSE}

The HIST2 transition fetches six tokens from the input places PRIORTY3, MAX PR2, PRIORTY4, MAX PR, HCNTR3, and PHIST2 and produces tokens to the output places PRIORTY3, PHIST2, MAX PR2, NEW DEST_PAUSE, MAX PR, and NEW DEST. We define four functions of prity(), prity2(), prityF(), and dstny to model the next destination after completing one epoch of the previous history.

Table 3 shows the declaration of polymorphic function dstny, which is used in the arcs expressions from the HIST2 transition to the NW DEST_PAUSE and NEW DEST output places.

**Table 3:** Declaration of functions used in an arc expression to model the next destination in Fig 8.

Line No.	Function
1.	val pr = 0.95;
2.	valpr2 = 0.70;
3.	val prF = 0.50;
4.	fun prity () = uniform (0.0,1.0) < = pr;
5.	fun prity2 () = uniform (0.0,1.0) < = pr2;
6.	fun prityF () = uniform (0.0,1.0) < = prF;
7.	fun dstny(id:NODE,dest:LCOOR fp:STRING,bb:INT,aa:INT, pdest:LCOOR, mdest:LCOOR) =
8.	(id,dest, fp, if aa+1 = 6 andalso bb = 0 then pdest else if aa+1 = 6 andalso bb > 0 andalso bb < 51
9.	then if prity () then pdest else [(real(discrete(0,99)), real(discrete(0,99)))]
10.	else if aa+1 = 6 andalso bb > = 51 then if prity2 () then if prityF () then mdest else pdest
11.	else [(real(discrete(0,99)), real(discrete(0,99)))]
12.	else [(real(discrete(0,99)), real(discrete(0,99)))]);

In order to model the history based destination, we first define the prity() function in line 4 of Table 3. When this function is called, it returns either *true* or *false* by evaluating the uniform (0.0,1.0) expression and produces a real number from the interval [0.0,1.0] where all numbers have the same selection probability. We define a constant <pr = 0.95> (line 1). If the number returned by the *uniform* function is less than or equal to the constant pr, then the prity() function is considered as *true;* otherwise, it is *false*. In other words, there is 95% chance of the occurrence of *true* and 5% chance of *false*: however, these parameters can be modified. The same logic can be applied to the prity2() and prityF() functions in the lines 5 and 6 of Table 3. To demonstrate a more realistic approach to find the destination, we selected the first fifty destinations to find five attraction points, out of which the first two attraction points are declared as hotspots (home and office). The destny polymorphic function has the objective to model the history based selection of the next destination of the MH. This procedure is defined in Algorithm-I. The destny function takes seven parameters as (id,dest,fp,bb,aa,pdest,mdest) and produces a four-tuple token, i.e., (id,home,fp,dest). The PAUSE MONITOR place is a monitoring place and holds the pause time intervals for future statistical analysis.

To define the pause time dependency, counter bb is modeled and shows the completed trips. To obtain realistic stable results, we implemented a built-in *discrete uniform distribution* function, and per policy, hotspots are given the pause time of (50–60), other attraction points of (20–40), and remaining new destination of (1–20). A user can modify these parameters. In our implementation, we applied the pause dependency policy after 60 completed trips. The OUT5 transition has one input and one output. This transition occurs when (bb < 60). The pause dependency is applied on its guard. In fact, the OUT PAUSE transition implements the pause dependency on its guard, and the next () recursive function is called to generate the pause time dependency. The PUSE DEPND port place is a monitoring place where the psudpnd() function with arguments (a,id,pl1,dest,tpr) has been implemented and returns the pause time delay based on the attraction point’s category.

Algorithm-I: History based destination

Input: B_bb,_ Counter used to find attraction points

        A_aa_, Counter for initial setup

        A_pdest_, Attraction point coordinates

        H_mdest_, Hotspot coordinates

        F_prity_. Probability function (95% chance to true)

        F_prity2_, Probability function (70% chance to true)

        F_prityF_, Probability function (50% chance to true)

Output D_Dest_, New destination based on previous movement history

1 ((aa+1 = = 6) and (aa+1 = = 6)-> pdest))

2 &

3 ((aa+1 = = 6) and (51<bb>0))

        a. (prity = = true-> pdest)

        b. else

        c. [(real(discrete(0,99)), real(discrete(0,99))]

4 &

5 ((aa+1 = = 6) and (bb > = 51))

        a. (prity2 = = true)

                i. (prityF = = true-> mdest)

                ii. else

                iii. (pdest)

        b. else

        c. [(real(discrete(0,99)), real(discrete(0,99))]

6 else

7 [(real(discrete(0,99)), real(discrete(0,99))].

### 2.3 Overview of the Occurrence Sequence

When the simulation starts, the only enabled transition for the proposed SHER CPN model is TimeofArival at page START. The model contains four parts: START, MOVE, HOTSPOTS, and PAUSE (see Fig 2). The MH identity with its availability time is positioned at the ND place and is represented by a token as *<1`1@0>*. This single tuple token specifies that an ID 1 is available at time 0. The TimeofArival transition generates two-tuple token representing MH-ID with arrival time, i.e., <*1`("MH-1"*,*6)@6>*. The MH2 place receives the token from the TimeofArival transition and enables the START transition, which is responsible to produce three-tuple token to represent MH-ID, home-grid-coordinates, and destination-grid-coordinates, i.e., <*1`(("MH-1"*,*6)*,*[(49*.*0*,*34*.*0)]*,*[(25*.*0*,*10*.*0)])@6>*. This token specifies that MH-1 wants to go from coordinates (49.0, 34.0) to coordinates (25.0, 10.0). The STA_HOM1 place that is the *port-socket* place between the START and SHER pages captures the token from the START transition and enables OUT transition on the ANGLE page. When the OUT transition occurs, it produces three tokens to the SLOP, TOTAL STPS, and MAX SPEED places. The five-tuple token, i.e., <1`(("MH-1",6),45.0,[(49.0,34.0)],[(49.0,34.0)],[(25.0,10.0)])> at the SLOPE place represents (MH, angle-to-move, Home-coordinates, Current-coordinates and Destination-coordinates). The TOTAL STPS place receives the two-tuple token <1`(("MH-1",6),34)> that contains the MH and total steps to reach the destination. The third output at the MAX SPEED place reflects four tuples as <1`(("MH-1",6),1,4,1)>. This token contains the MH, current speed, maximum speed, and current step number information. From these three outputs, we can conclude that OUT transition produces MH-1 from home coordinates (49.0, 34.0) to the destination coordinates (25.0, 10.0) with the moving angle of 45^o^ and it will take 34 single steps, and the selected speed for this epoch is four. The QURDNT transition delivers final angle with respect to four quadrants, and in this case the destination falls in the fourth quadrant. Therefore, the final angle is 225^o^ when the token is at the QURDNT SLOP place, i.e., <1`(("MH-1",6),225.0,[(49.0,34.0)],[(49.0,34.0)],[(25.0,10.0)])>. The READY transition takes all basic information from the five input places and produces the following eleven-tuple token at the NOW MOVE output place:<1`(("MH-1",6),1,[(49.0,34.0)],[(49.0,34.0)],[(25.0,10.0)],225.0,4,1,34,1,1.0)>.

**Fig 10:**
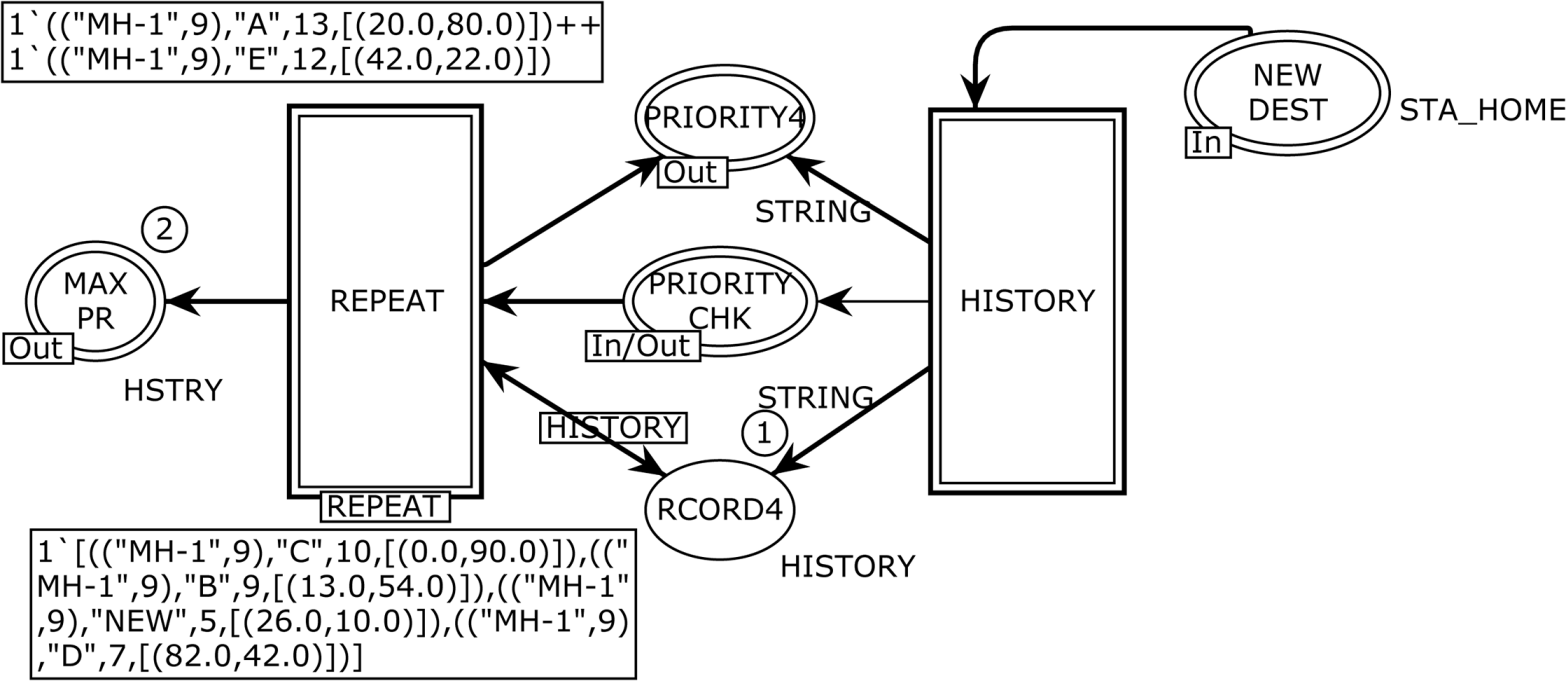
The HOTSPOT module after 2300 steps which reflects two hotspots “A” and “E.”

The contents of this token are MH, trip number, home coordinates, current coordinates, destination coordinates, angle to move, maximum speed, current speed, total steps, current step number, and tracking counter. Now, the RUN transition at the MOVE page becomes enabled by the NOW MOVE input place. It takes three inputs and produces an eleven-tuple token to the NOW MOVE place after incrementing one step in the given binding. The go function implemented on the arc from the RUN transition to the NOW MOVE place elegantly takes steps towards the destination by calling the slow function to gradually increase or decrees the speed. The EVERY MOVE place is a monitoring place, which keeps the record of every step, and in current epoch, the MH has reached its destination in twelve steps <1`(12,("MH-1",6),[(25.95,10.95)],[(25.0,10.0)])>. After successful completion of the first trip, the RUN transition sends a token to the NEW DEST output place, which enables the PRITY transition at the HISTORY page. The PRITY transition produces a token at the PHIST place after mentioning the first attraction point “A” with coordinates. Now the token can be seen as <1`(("MH-1",6),"A",[(25.0,10.0)])>. The CHKHIST transition fetches the token from the PHIST place and forwards it to the HSTY3 place after keeping the record back at the PHIST place. The UPHSTRY transition enables after the token is at the HSTY3 place, and when it occurs, it leads the posting of the history based token at the RCORD3 place and one token at the PRIORITY4 output place reflecting the attraction point “A”. Now control moves to the PAUSE page by having a token at the PRIORITY4 input place. The objective of the HIST2 transition is to select a new destination; hence, when it occurs, it produces the token <1`(("MH-1",6),[(25.0,10.0)],"A",[(54.0,8.0)])@18 > at the NW DEST place. Here, we can see that the previous destination is now converted to home and a new destination is presented as [(54.0, 8.0)]). The OUT5 transition fetches a token from the NW DEST place (see Fig 9) and sends it to the STA_HOM1 place at the SHER page (see Fig 2). Thus, the SHER model represents acyclic non-terminating behavior. In order to obtain a complete list of five attraction points, Fig 10 demonstrates anther execution of the SHER model, where we repeated the cyclic firing sequence of the transitions 50 times. The marking of the HOTSPOT module after 2300 steps represents two hotspots and three attraction points. It can be seen that the MAX PR place indicates two hotspots: <1`(("MH-1",9),"A",13,[(20.0,80.0)])> and <1`(("MH-1",9),"E",12,[(42.0,22.0)]) >. These two attraction points are declared as hotspots on the basis of maximum number of visits and in the given case, 13 visits are at point “A” and 12 visits are at point “E”. The RCORD4 place indicates three attraction points with visit frequencies of 10, 9, and 7 as <1`(("MH-1",9),"C",10,[(0.0,90.0)])>, <1`(("MH-1",9),"B",9,[(13.0,54.0)])>, and <1`(("MH-1",9), "D",7,[(82.0,42.0)])>. In addition, five visits are towards any other ordinary places.

## 3 Simulation Analysis and Discussion

**Fig 11:**
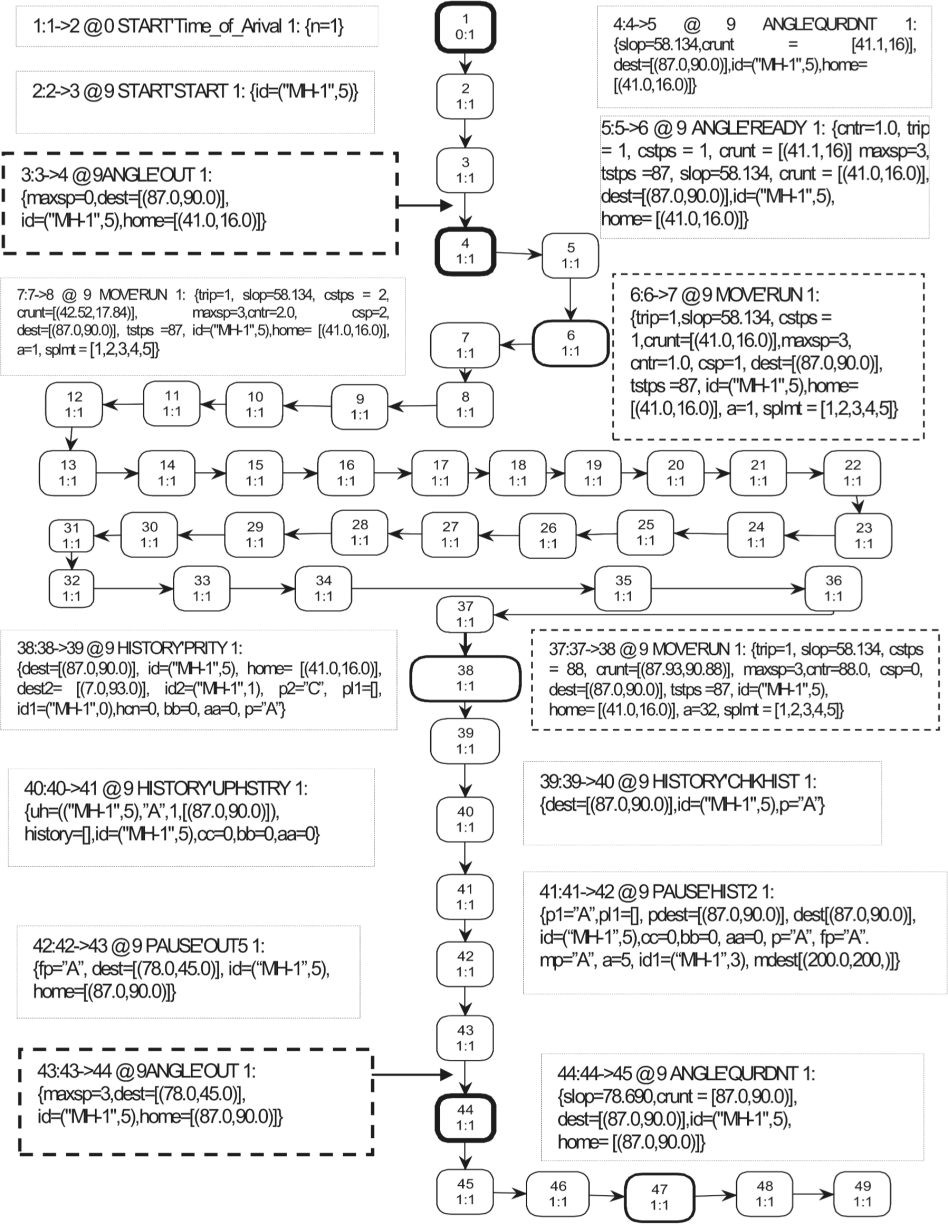
Partial state space representing the occurrence graph of the SHER mobility model.

In order to simulate and verify the SHER mobility model, we used the CPN state space tool and implemented a state space construction algorithm. The state space analysis allowed us to analyze all the standard behavioral properties of the model such as *boundedness*, *reachability*, *liveness*, *home* and *fairness*. For simplify the analysis and monitor the behavior of the proposed model and its movement patterns, the state space tool’s options were fixed in order to partially generate a simulation up to 1000 sec. Table 4 shows the initial part of the standard state space report for the movement of one MH and its statistics. It specifies that the O-graph (occurrence graph) has 42244 nodes and 57145 arcs, and the Strongly Connected Graphs (SSG) immediately reveals that cycles exist, and the model has an infinite occurrences sequences and does not terminate. It took four seconds to calculate the SSG. The MH moved in communication range without any restriction. Fig 11 depicts the partial state space that represents the O-graph.

**Table 4:** State space report–statistics.

Line No.	State Space	Scg Graph
1.	Nodes:42244	Nodes:42244
2.	Arcs: 57145	Arcs: 57145
3.	Secs: 1000	Secs: 4
4.	Status: Partial	

### 3.1 Reachability Graph

Reachability analysis techniques are widely used in formal verification of the concurrent systems and protocols [39][40]. As shown in Fig 11, the initial fragment of the O-graph has 49 nodes and 48 arcs, and it can be seen that each node (marking) is inscribed with three integers where the topmost integer in the node of the O-graph indicates the node number and the other two separated by colon (:) represent the total number of successor and predecessors. The state space in Fig 11 is represented by the directed graph where each node shows reachable marking and an arc illustrates the occurrence binding element. There is a single one to one (1:1) sequence of the occurrence without overtaking. The rectangular box positioned next to the node reflects the binding information. The arc binding (thick dashed border) of node 4 is <3:3->4 @ 9 ANGLE'OUT 1: {maxsp = 0,dest = [(87.0,90.0)], id = ("MH-1",5),home = [(41.0,16.0)]}> and the binding for node 44 is <43:43->44 @ 9 ANGLE'OUT 1: {maxsp = 3,dest = [(78.0,45.0)],id = ("MH-1",5),home = [(87.0,90.0)]}>. These bindings provides the evidence of a cyclic non-terminating behavior of the SHER model. The structure of node 4 and 44 is the same; however, there are variable parameters according to the destination coordinates of the MH. The first two nodes (1 and 2) represent the occurrence of transitions that takes place from the START module, then control is transferred to the ANGLE module (node 3, 4, and 5). Next, the MH travels to the destination coordinates via the MOVE sub-page, and 32 nodes (from 6 to 37) represent the traveling pattern of the MH from home to destination. In each epoch, these nodes are changed according to the destination distance from home. When MH finishes the first trip, the HISTORY submodule is enabled. Moreover, three nodes (38 to 40) in the O-graph represents the behavior of this sub-page. We can proceed analogously in the PAUSE module where three nodes (i.e., 41, 42, and 43) define the pause time structure after completion of the first epoch. Next, the SHER model repeats the process for the next destination from the ANGLE module. Node 44 represents the start point of the repeating process (the OUT transition of the ANGLE module).

### 3.2 Boundedness Properties

Upper and lower bounds property specifies the maximum and minimum number of tokens that can reside on a specific place in any reachable marking. It is important to determine whether the proposed model prevents an overflow. A net is said to be *k-bounded*, if any place contains less than or equal to *k* tokens where *k* is any non-negative integer. If the bound for the number of tokens is equal to one (1-bounded), then this net is *safe*. Table 5 shows part of the state space report for the *boundedness* properties. The CPN model has 52 places. Among these, 34 places have one *best upper integer bound* (1-bounded), and this means that the proposed model is *safe*. The PAUSE'PAUSE_MONITOR, SHER'MONITOR, SHER'PUSE_DEPND, and SHER'EVERY_MOVE places are *unbounded*, and have *best upper integer bound* as 7, 5, 25, and 65 respectively. In fact, these places are monitoring places with the purpose to keep track of every step and their fetched results are used in statistical analysis. If these places are removed, there will be no effect on the model’s execution and performance. The PAUSE'PRIORTY3 place has the *best upper* and *lower* bound that are equal to five. This implies that this place always contains equal number of tokens in any reachable marking. The PRIORTY3 place is used to model five attraction points.

**Table 5:** State space report—Boundedness properties and integer bounds.

Best Integer Bounds	Upper	Lower	Best Integer Bounds	Upper	Lower
**ANGLE'CONTER**	1	1	PAUSE'NW_DEST	1	0
**ANGLE'MAX_SPEED**	1	0	PAUSE'NW_DEST_PAUSE	1	0
**ANGLE'OK**	1	1	PAUSE'NW_DEST__PAUSE2	1	0
**ANGLE'QURDNT_SLOPE**	1	0	PAUSE'PAUSE_MONITOR	7	0
**ANGLE'SLOPE**	1	0	PAUSE'PHIST2	5	0
**ANGLE'TOTAL_STPS**	1	0	PAUSE'PRIORTY3	5	5
**ANGLE'VLCTY_DEPND1**	1	0	PAUSE'PRITY_PLACES2	1	1
**HISTORY'HCNTR**	2	0	PAUSE'PS_CNTR	1	1
**HISTORY'HCNTR2**	2	0	PAUSE'PS_CNTR2	1	0
**HISTORY'HCNTR4**	1	1	REPEAT'BCNTR	1	0
**HISTORY'HSTY3**	2	0	REPEAT'BCNTR2	1	0
**HISTORY'PHIST**	5	0	REPEAT'MAXP	1	0
**HISTORY'PHIST5**	1	1	REPEAT'PR_INT	1	0
**HISTORY'PHIST8**	1	0	REPEAT'RCORD	1	0
**HISTORY'PHIST9**	1	0	REPEAT'SORTED	1	1
**HISTORY'PRIORITY**	2	0	REPEAT'SRTCNTR	1	1
**HISTORY'PRIORTY2**	6	1	SHER'MAX_PR	2	1
**HISTORY'PRITY_PLACES**	1	1	SHER'MONITOR	5	0
**HISTORY'RCORD3**	1	1	SHER'ND	1	0
**HOTSPOTS'RCORD4**	1	0	SHER'NEW_DEST	2	0
**MOVE'CNTR**	1	1	SHER'PRIORITY4	2	0
**MOVE'NOW_MOVE**	2	0	SHER'PRIORITY_CHK	1	0
**MOVE'TSP**	1	1	SHER'STA__HOM1	1	0
**PAUSE'HCNTR3**	2	0	START'MH2	1	0
**PAUSE'MAX_PR2**	1	1	SHER'PUSE_DEPND	25	0
**START'VLCTY_DEPND**	1	0	SHER'EVERY_MOVE	65	0

**Fig 12:**
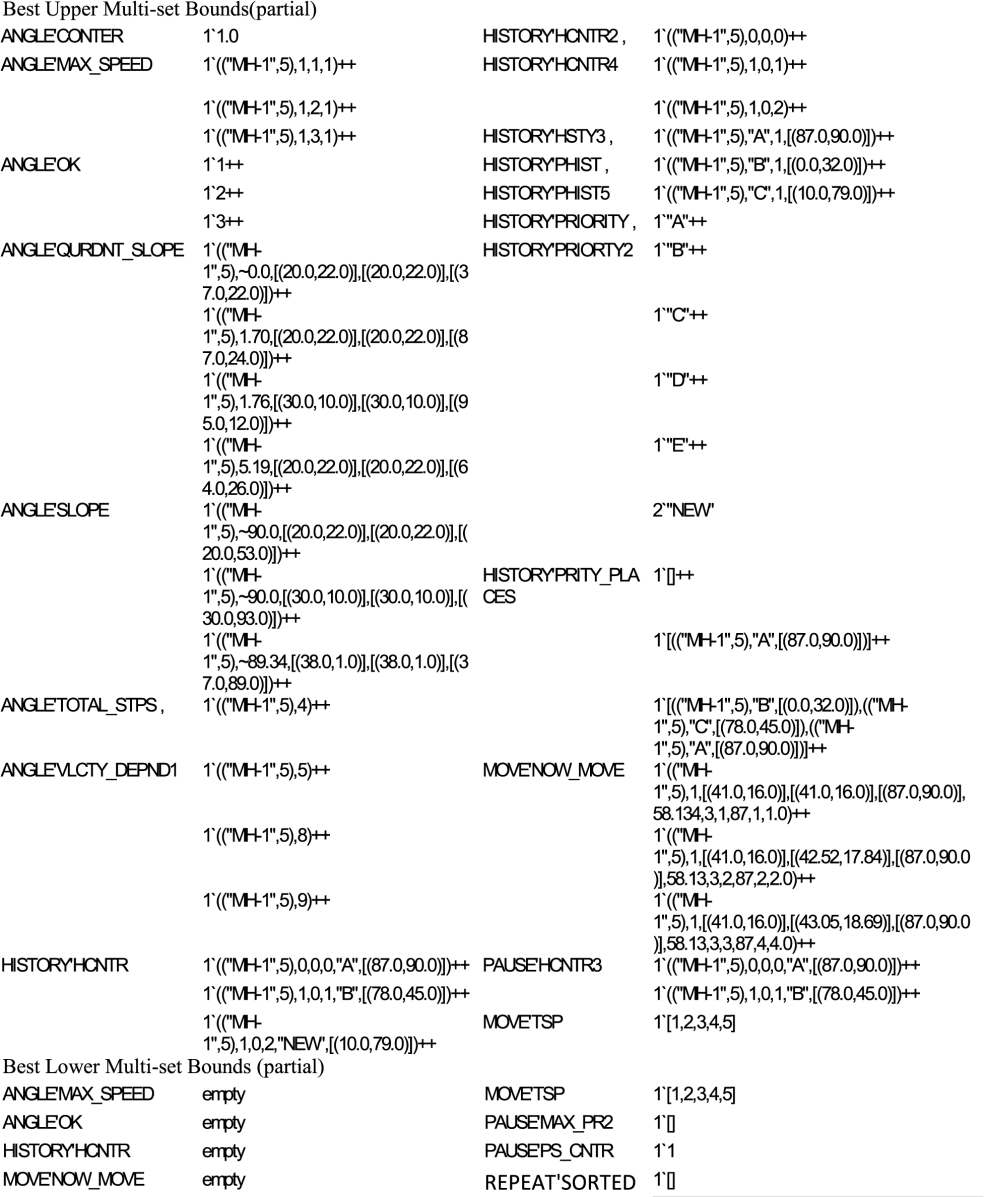
State space report-multi-set bounds.

Moreover, we considered only the maximum and minimum number of tokens that can reside in a specific place ignoring the token types. Fig 12 illustrates the best upper and lower multi-set bounds for both quantity and the color of residing tokens. To save the space, we mentioned the multi-set bounds and number of tokens of some places. As expected, these multi-sets are an example that the MAX_SPEED place at the ANGLE page specifies three 4-tuple tokens and in the third tuple the speed of the MH gradually increases. The OK place at the ANGLE page shows the counter for the number of trips, which increases in every turn. The PRIORITY place on the HISTORY page specifies string type tokens that represent five attraction points. The PRITY_PLACES place at the HISTORY page shows one token as empty list, then this list increases by adding attraction points and their coordinates. The TSP place at the MOVE page contains a single token in both *upper* and *lower* bound cases and it remains unchanged during simulation.

### 3.3 Deadlock freedom

**Fig 13:**
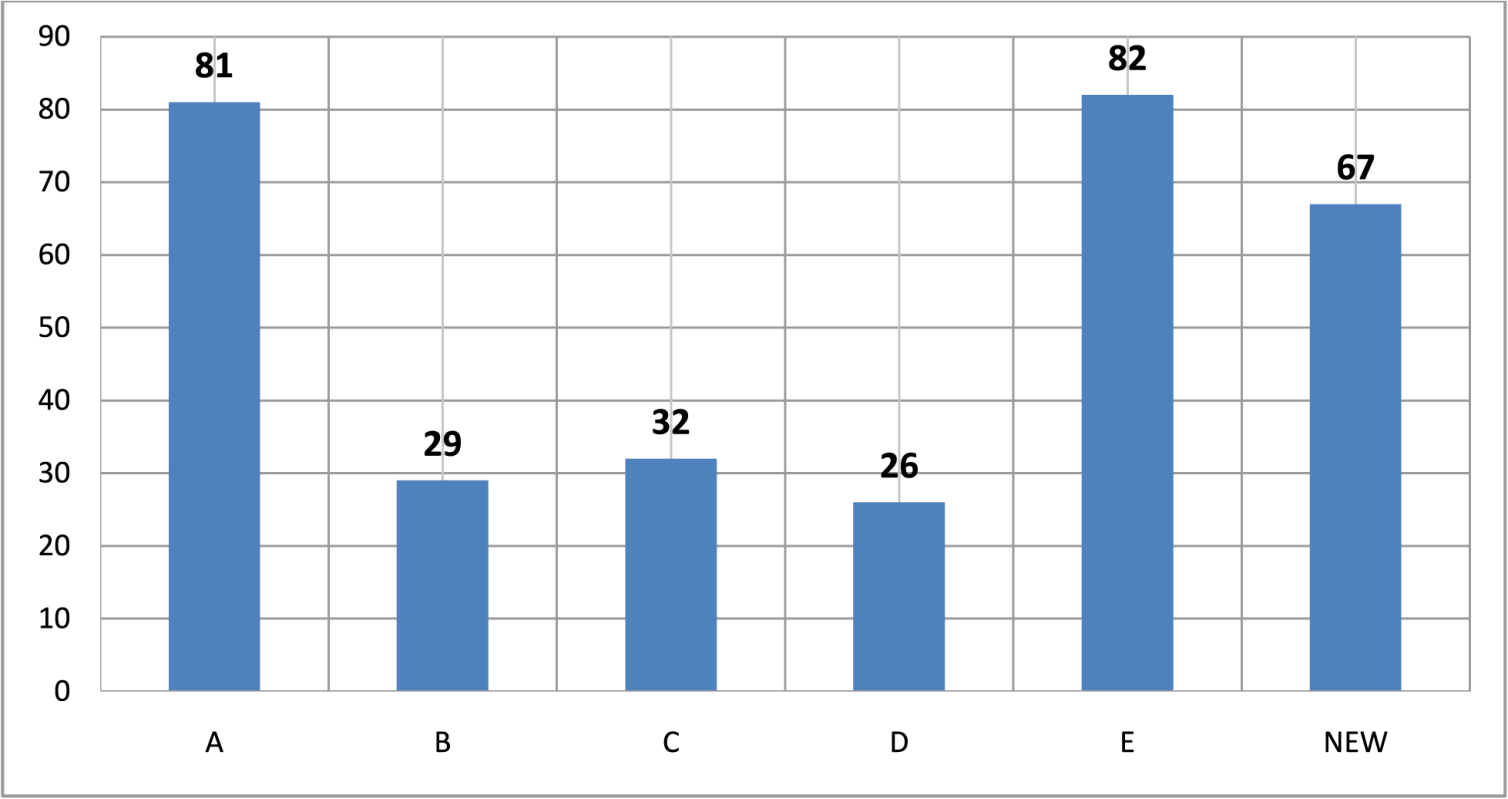
Hotspots, attraction points vs number of trips.

**Fig 14:**
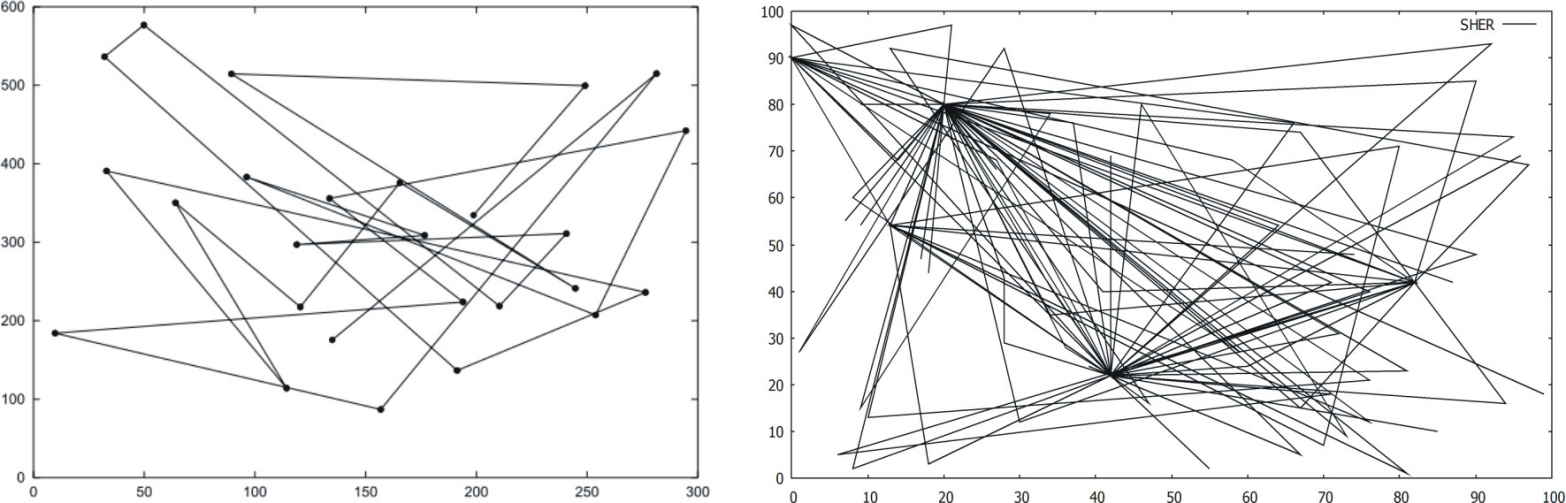
Traveling pattern of a mobile node (MN) using the RWP mobility model [12], Cyclic history based behaviour of the SHER mobility model.

**Fig 15:**
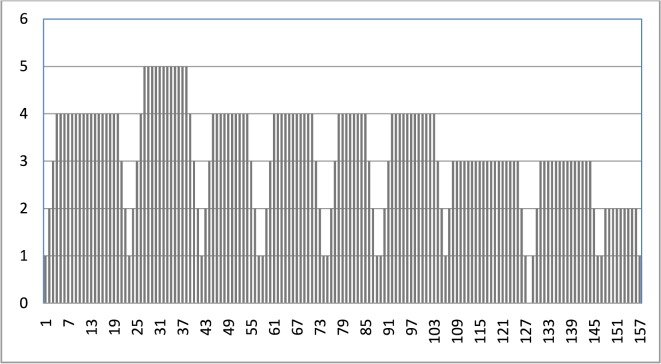
Slow start, stop, and velocity dependency.

**Fig 16:**
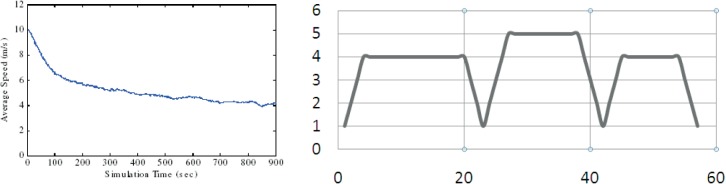
Average speed decay (speed = (0, 20) m/s, pause = 0 sec)[17], Cyclic history based behaviour of the SHER mobility model.

To determine the failure cause of the model, Holzmann [41][42] presented three prominent properties: *deadlocks* are the cause when the protocols are in waiting state for occurrence of conditions that can never be fulfilled and leads to deadlock of the model; *livelocks* represent the situation when every process constantly changes with regard to one another without making any effective progress. Finally, *improper termination* is the situation when the protocol accomplishes without satisfying the termination conditions. Our proposed model is deadlock free and the state space report in Table 6 specifies that there are 15671 dead markings including [42244,42243,42242,42241,42240,…]. There are 42244 dead marking because we fixed the state space tool to generate state space report up to 1000 sec for the purpose of simplicity; hence, the total generated nodes are 42244 (see Table 4). Our proposed SHER model satisfies all the termination specifications and Figs 13 to 16 illustrate these conditions.

**Table 6:** State space report-home and liveness properties.

Sr. No.	Properties	Sub-Properties	Description
**1.**	**Home Properties**	Home Markings	Initial Marking is not a home marking
**2.**	**Liveness Properties**	Dead Markings	15671 [42244,42243,42242,42241, 42240,…]
		Dead Transition Instances	HISTORY'H2ND 1
			HISTORY'H3ND 1
			PAUSE'OUT_PAUSE 1
			PAUSE'PRE_OUTPAUSE 1
			REPEAT'FMAX 1
			REPEAT'MAX_PRITY 1
			REPEAT'PRIORITY_CNTR 1
			REPEAT'SORT 1
		Live Transition Instances	None
**3.**	**Fairness Properties**	Fairness	No infinite occurrence sequences.

The home properties in Table 6 specifies that initial marking is not home marking. However, Fig 11 illustrates that we can repeat the process (see the bold node 4 and 44 of Fig 11). In markings 4 and 44, the tuples, color sets, and variables can never be changed; however, values of variables can be changed according to the current scenario. The *liveness* property ensures that a transition *t* can be fired again and is stronger than the absence of dead transition. Similarly, the home property specifies that a marking *m* can always be reached again during the model execution. The proposed SHER mobility model has *partial home-marking* and *partial liveness* as presented in Fig 11.

### 3.4 Livelock Deadlock

As stated above *livelocks* are special cases for resource starvation when a specific module or process does not progress. Our implementation of the SHER model is without any *livelock*. Fig 11 provides the relevant supporting evidence for the node’s one to one (1:1) relation.

### 3.5 Termination

Improper termination is another prominent property used for verification and specifies whether a proposed model is *terminating* or *not*. A termination state reflects a marking when no further transition is enabled or the model has finite runs. The proposed SHER mobility model has infinite runs; therefore, it is *not terminating* because the model has a cyclic structural behavior. Fig 11 illustrates the O-graph generated from the CPN model where bold *nodes* (see 4 and 44) *verifies* acyclic *non terminating* property of CPN model.

### 3.6 Dead Transition

A transition *t* is called a dead transition in the marking *m* if it can never become enabled in any reachable marking from *m*_*0*_ during execution. This means that every transition should be occurred at least once on every run. Our proposed model does not have any dead transition (see Fig 11). This can also be verified by implementing the following query that shows an empty list.

ListDeadTIs()→val it[]≕TI.TransInst list

### 3.7 History Based Movement Patterns without Border Effect Problem

After successful termination of 1000 sec simulation, the data shows 317 completed trips for the statistical analysis. We obtained two hotspots [“A”, (20,80)] and[“E”,(42,22)] with the frequency of visits equal to 81 and 82, three attraction points [“B”,(13,54)], [“C”,(0,90)], and [“D”,(82,42)] with 29, 32, and 26 number of visits respectively. There are also 67 trips to a “NEW” (anonymous) locations. Fig 13 yields the frequency of visits while the graphical representation of these trips is illustrated in Fig 14.

The broad criticism about the RWP mobility model is that it generates memory-less mobility patterns, because it retains no knowledge to its past locations. This behavior is depicted in the left part of Fig 14 [12]. In addition, the researchers investigated that the RWP mobility model suffers from the border effect phenomena. The proposed SHER mobility model precisely avoids such problems that are discussed in the literature. The traveling pattern generated by the SHER, which is depicted in the right part of Fig 14, exhibits its improved behaviors.

### 3.8 Slow Start, Stop, and Velocity Dependency

Fig 15 shows more a realistic mobility pattern through slow start, stop, and velocity dependency algorithms that are implemented in the SHER mobility model. It can be seen that there were nine completed epochs, where the first trip had a maximum speed equal to four and in the second trip, the speed was increased by one and the MH completed the trip with the speed of five. Thereafter, speed was gradually decreased by one in the third trip and resulted in a maximum speed of four. In the 4^th^, 5^th^, and 6^th^ trip, speed remained stable at four. Later on, in the 7^th^ trip, speed decreased again and was three. In the 8^th^ trip, it remained constant. Finally, in the last trip, the speed decreased by one and became two.

The slow start and stop procedure was intelligently adopted by the SHER model and is depicted in Fig 15. It is evident that the MH started each epoch with speed of one and gradually increased up to the maximum speed. After achieving its maximum limit, the MH traveled towards the destination with this speed and gradually decreased speed up to one before its stop. The same process was repeated until simulation stopped.

### 3.9 No Speed Decay Problem

Another problem mentioned in the literature about the random models is the *“speed decay”* issue[17] when instantaneous average speed of the MH is consistently decreasing (see left part of Fig 16). The proposed SHER mobility model exhibited an MH speed without *speed decay* problem. The right part of Fig 16 demonstrates this behavior. It is pertinent to mention that after taking the first step with slow start, MH gradually achieved steady speed, then remained constant, and gradually decreased near destination. To elaborate further, Fig 16 presents comparison between the SHER and RWP mobility models.

### 3.10 Pause Time Dependency

**Fig 17:**
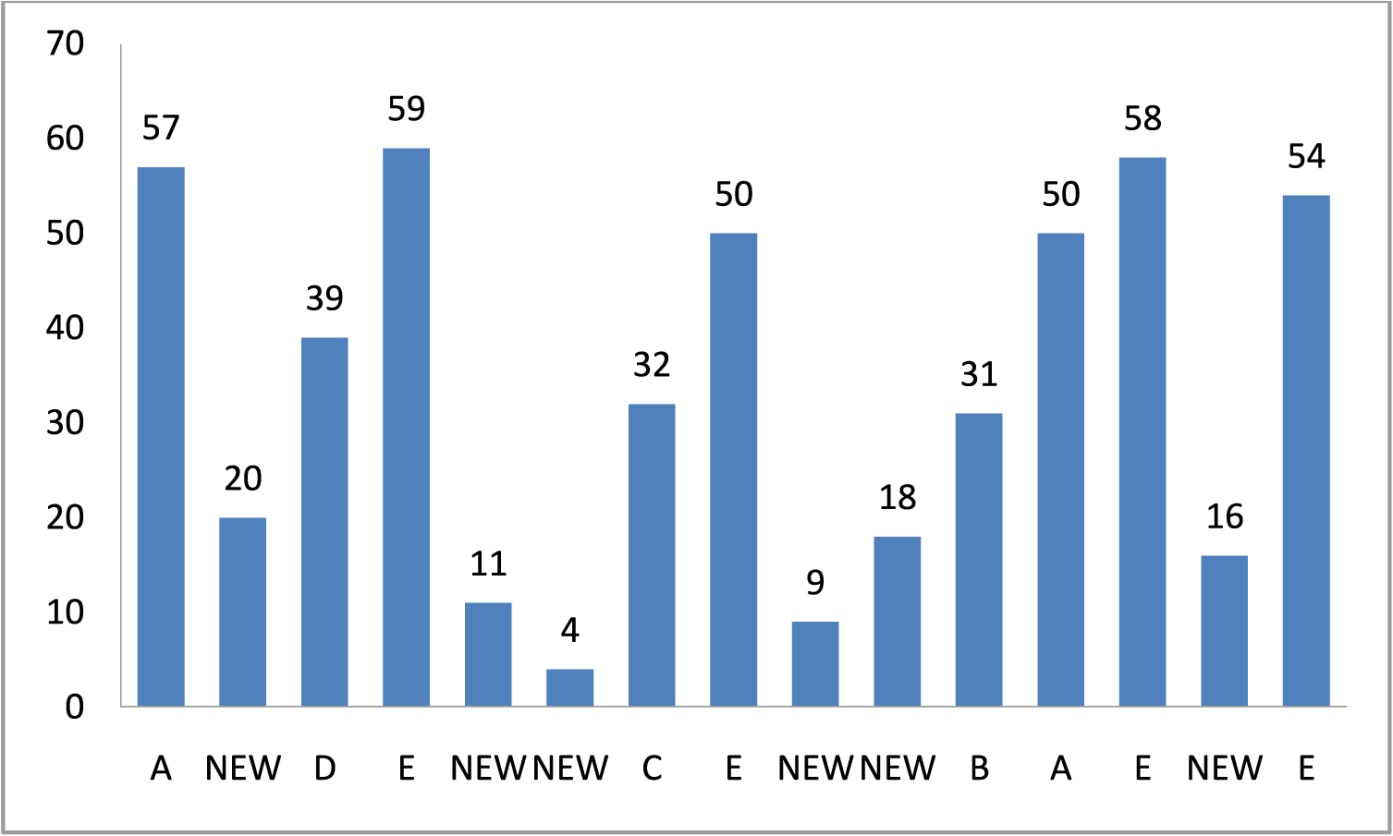
Pause time intervals of 15 epochs.

Fig 17 demonstrates the pause time dependency generated by the SHER mobility model. As narrated in Section 3.3, the pause time dependency is based on the destination type. We modeled the pause time, i.e., (50–60) for hotspots, (20–40) for attraction points, and (1–20) for “NEW” destinations. The pause time dependent algorithm randomly selected the pause time of an MH based on the explained criteria. There are 15 slots for the pause time shown in Fig 17, and we can recognize six hotspots, three attraction points, and six “NEW” (anonymous) destinations.

## Conclusion

The world of wireless network communication is rapidly changing with recent innovations like WMNs. To the best of our knowledge, we are the first research group to present the SHER mobility model as explained in the introduction in order to remove the six key issues, i.e., *sudden stops*, *memory less movements*, *border effect*, *temporal dependency of velocity*, *pause time dependency*, *and speed decay* from the existing random mobility models. In this study, a timed-hierarchical CPN based formal model was developed that was created based on the social aspects of human mobility to produce realistic history based movement patterns. Most of the existing researches are based on simulators such as NS-2, OPNET, and QualNet, while our work opens a new formal modeling based paradigm of research. Extensive simulation experiments were conducted in order to verify the validity of the proposed model. Our results confirmed that the proposed SHER mobility model exhibited a reliable approximation of realism. The proposed model was suitable to develop different topologies and produced diverse trajectories. These patterns was further implemented to remove the mobility management performance issues. The model was easily altered and broadened to capture complex scenarios. In addition, the model was suitable for newcomers to the research community due to its simplicity. As part of our future work, we propose a dynamic mobility model where a token represents obstacles. At this stage, the SHER mobility model produces mobility patterns of a single user while in the next version, we will enhance the capabilities of the SHER to exhibit group based movement patterns. Further research may be devoted to develop realistic mobility models.
